# Quantification of evolved DNA-editing enzymes at scale with DEQSeq

**DOI:** 10.1186/s13059-023-03097-3

**Published:** 2023-11-06

**Authors:** Lukas Theo Schmitt, Aksana Schneider, Jonas Posorski, Felix Lansing, Milica Jelicic, Manavi Jain, Shady Sayed, Frank Buchholz, Duran Sürün

**Affiliations:** 1 Medical Faculty and University Hospital Carl Gustav Carus, UCC Section Medical Systems Biology, Dresden, TU Dresden 01307 Germany; 2 Present Address: Seamless Therapeutics GmbH, Tatzberg 47/49, 01307 Dresden, Germany

**Keywords:** Deep sequencing, Directed evolution, Site-specific recombination, Cre recombinase, CRISPR, Base editing, Cas12f

## Abstract

We introduce DEQSeq, a nanopore sequencing approach that rationalizes the selection of favorable genome editing enzymes from directed molecular evolution experiments. With the ability to capture full-length sequences, editing efficiencies, and specificities from thousands of evolved enzymes simultaneously, DEQSeq streamlines the process of identifying the most valuable variants for further study and application. We apply DEQSeq to evolved libraries of Cas12f-ABEs and designer-recombinases, identifying variants with improved properties for future applications. Our results demonstrate that DEQSeq is a powerful tool for accelerating enzyme discovery and advancing genome editing research.

**Supplementary Information:**

The online version contains supplementary material available at 10.1186/s13059-023-03097-3.

## Background

Directed evolution applies the principles of Darwinian evolution to the laboratory to improve protein features [1, 2]. During rounds of mutagenesis and selection, large gene variant libraries (~ 10^5^– ~ 10^8^) are produced [3–5]. Screening libraries to identify efficient variants is conventionally a manual process that is labor, resource, and time intensive. Furthermore, the number of variants that can be tested is limited, reducing the probability of identifying optimal variants. A high-throughput method for comparing large sets of enzymes would be desirable.

Indeed, a number of applications have been developed for high-throughput screening of enzyme variants. CombiSEAL [6], for instance, allows screening of combinations of defined mutations, but it is not well suited for analyzing evolution products. Evoracle [4] on the other hand, is suitable for this purpose, because it infers fitness and sequence composition of genes using sequence data of multiple evolution cycles. However, it can not be used for analyzing variants on multiple target sites. evSeq [7] is a microplate-based approach where variant phenotypes can be screened separately and the genes are sequenced as pools with barcodes. While this allows for a lot of flexibility, the microplate format limits the total number of variants that can be screened and may require automated liquid handling to be efficient. Fully high-throughput is UMIC-seq [8], as it employs random barcoding and nanopore sequencing to analyze a large amount of variants, but no phenotypic information is acquired. To conclude, previously published methods have limitations when it comes to efficient linkage of genotype and phenotype information on a large scale.

Here, we present DEQSeq (DNA Editing Quantification Sequencing), a high-throughput screening platform that enables the characterization of thousands of DNA editing enzyme variants on multiple target sites. The approach utilizes nanopore technology for sequencing of full-length enzyme variants at fast turn-around times. Through clustering of unique molecular identifiers (UMI), a highly accurate consensus sequence of the enzyme variants is generated [8, 9]. By simultaneously capturing the target site sequence with the enzyme sequence on the same DNA fragment, the DNA editing rate for each enzyme variant can be quantified. We demonstrate the applicability of this platform on two different DNA editing systems, namely designer-recombinases [5, 10–15] and evolved Cas12f-derived [16–19] mini-ABEs.

## Results

**Fig. 1:**
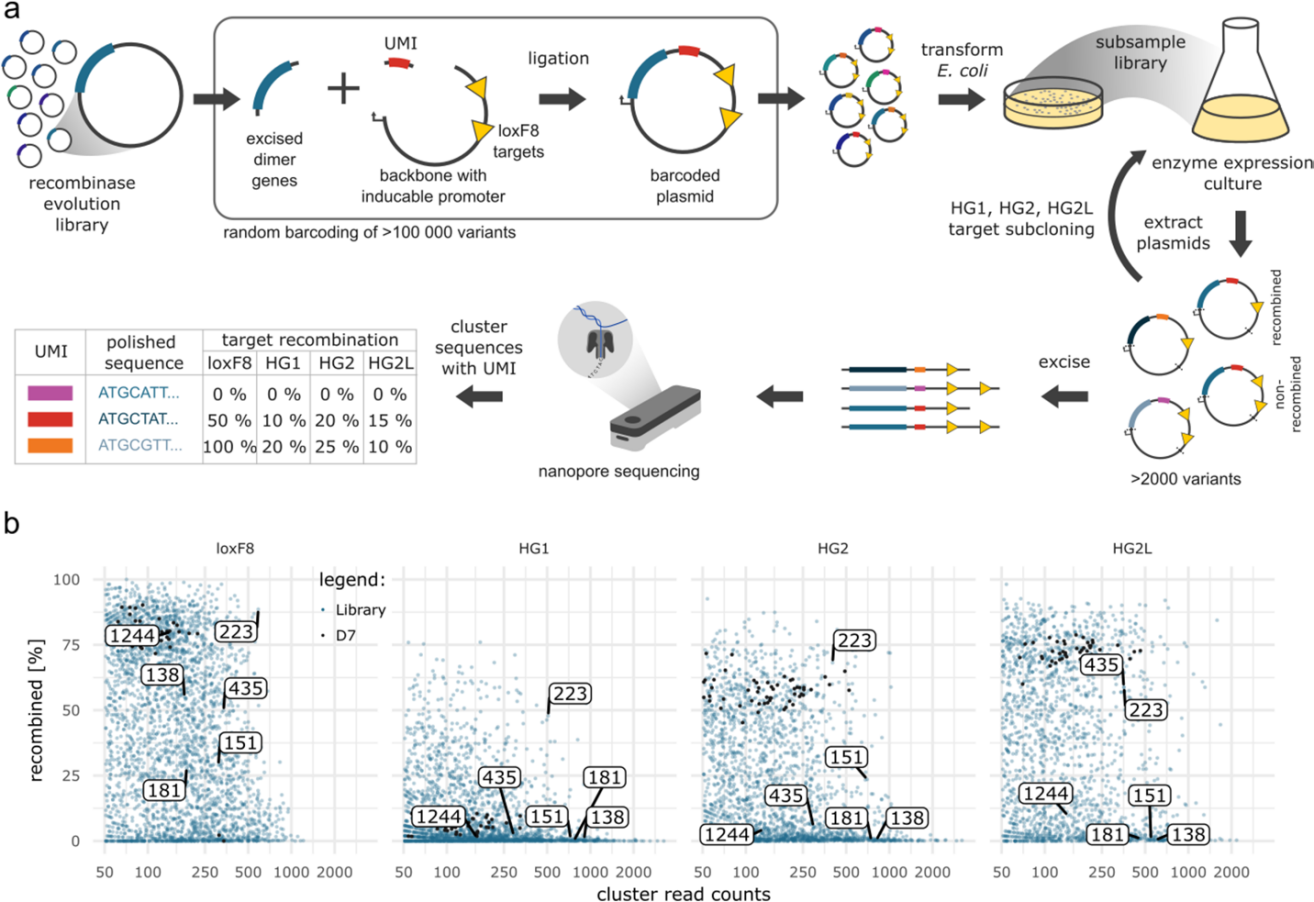
DEQSeq identifies favorable designer-recombinases. **a** Workflow overview. Evolved recombinases are cloned together with a unique molecular identifier (UMI) into a vector containing loxF8 target sites. *E. coli* are transformed with the barcoded plasmids. The number of transformed bacteria is determined by counting colonies on agar plates with an antibiotic matching the resistance gene on the plasmids. Calculated from the transformation efficiency, a defined number of transformed bacteria is cultured. Recombinase expression is induced by the addition of L-arabinose, which may result in the recombination of the target sites on the plasmid. The plasmid DNA is isolated and the recombinase genes and their UMIs are subcloned into pEVOs with different target sites (HG1, HG2, and HG2L) and cultured as before. From all the cultured plasmids, the region containing the gene, the UMI, and the target sites are excised and sequenced with nanopore sequencing. The sequences of the UMIs are computationally clustered based on similarity, resulting in groups of sequences corresponding to the different variants. From these groups, the recombination events on the different target sites are counted and accurate recombinase sequences are generated. **b** DEQSeq screen of loxF8 recombinases on the four target sites as indicated (loxF8, HG1, HG2, HG2L). UMI-clusters containing evolved recombinases are indicated as blue points and D7 control clusters are indicated in black. Marked with numbers are recombinase variants that were further evaluated. Enzyme expression was induced with 1 µg/ml L-arabinose on loxF8 and 100 µg/ml L-arabinose on all other target sites

We first performed DEQSeq on a library of evolved site-specific recombinase pairs that target a sequence in the human FactorVIII gene (loxF8; Fig. 1a). As a control we included the D7 variant pair that had been identified by picking and evaluating 96 random variants from the final library [10]. The aim of the screen was to identify variants that have lower off-target activity compared to D7, while maintaining similar on-target activity. Therefore, additionally to the loxF8 target site, we also screened the library of evolved recombinases simultaneously on 3 off-targets that are recognized by D7 (HG1, HG2, and HG2L) [10] (Fig. 1b). In total the screen yielded 2515 UMI-clusters with 50 or more reads, from which we identified 53 clusters as D7 control. Analysis of the polished D7 recombinase sequences revealed no sequence errors, indicating an accuracy of close to 100%. Median recombination rates of D7 were 80.2% on its intended target, 5.8% on HG1, 57.7% on HG2, and 72.6% on HG2L (Additional file 1: Fig. S1a). Of the 2476 non-D7 clusters we identified 70 clusters that had less than 10% off-target activity on the 3 off-targets and more than 25% activity on the on-target (Additional file 1: Fig. S1b).

**Fig. 2:**
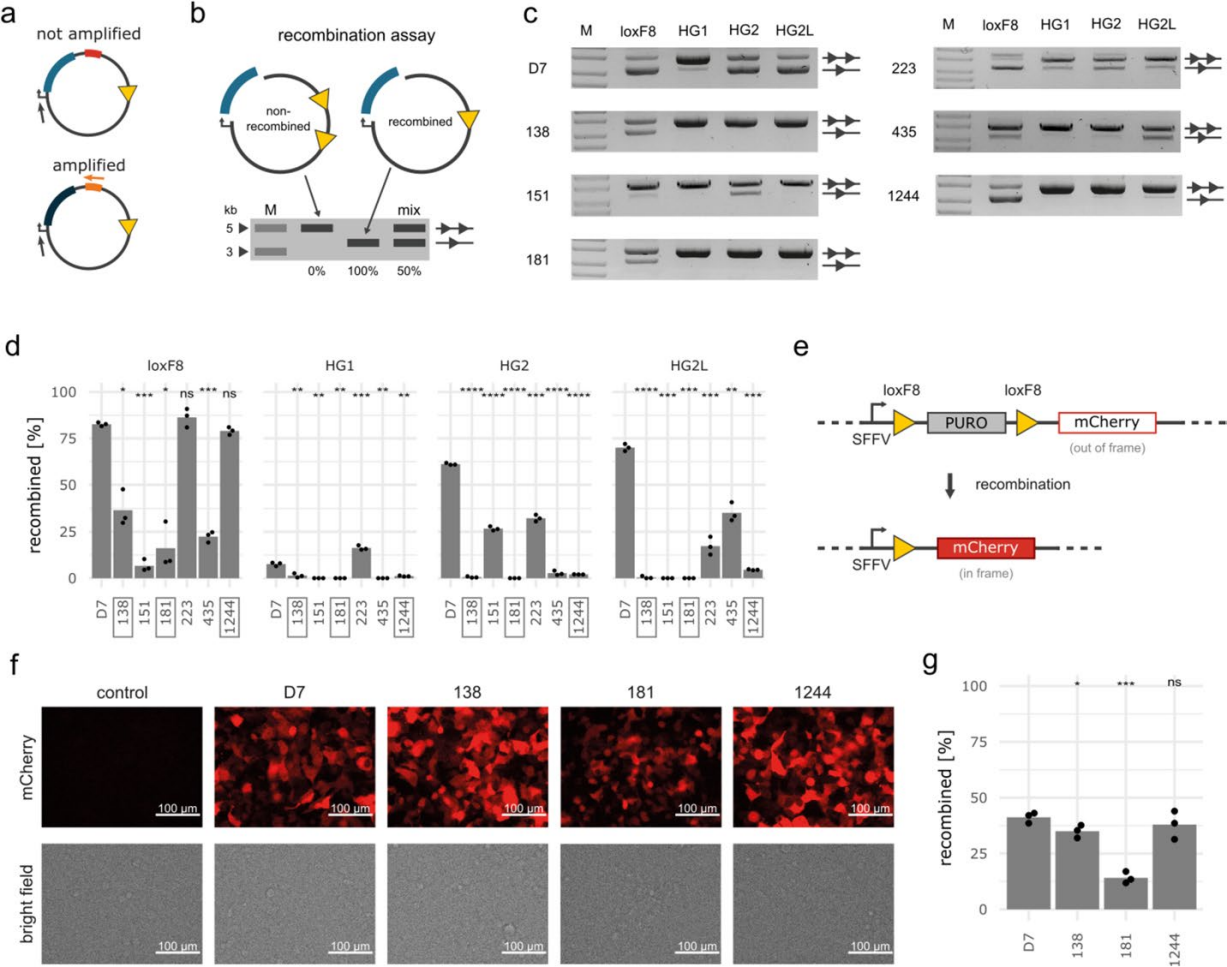
Validation of selected recombinase variants. **a** Schematic illustration of targeted PCR amplification of desired variants. A universal primer binds upstream of the recombinase genes (gray arrow) and a UMI-specific primer only binds to the UMI of the variant of interest. A subsequent PCR reaction only amplifies the desired variant. **b** Schematic illustration of the recombinase plasmid assay. The running properties on an agarose gel of a non-recombined plasmid (two triangles) and a fully recombined plasmid (one triangle) are depicted. The band intensities can be used to calculate recombination efficiencies (in %) as illustrated in the “Mix” lane (50%). M = DNA ladder with indicated fragment sizes (kb). **c** Agarose gels and **d** quantification of recombination products of clusters tested with the plasmid assay (*n* = 3). The assay was performed with the same induction levels as for the screen. Statistical results from t-tests comparing the variants to D7 are included above the bars. Boxed variants display low off-target recombination. **e** Schematic illustration of the integrated reporter construct in HEK293T cells. Expression of mCherry is blocked by the puromycin (PURO) gene. After recombination the PURO cassette is excised, leading to expression of the mCherry gene. **f** Fluorescent and brightfield images and **g** flow cytometry quantification of recombination 3 days after transfection of HEK293T.^loxF8^ reporter cells with the indicated recombinases. Statistical results from *t*-tests comparing the variants to D7 are included above the bars in **d** and **g**. *P*-values: “ns” not significant, * *p* < 0.05, ** *p* < 0.01, *** p < 0.001, **** *p* < 0.0001

To validate the DEQSeq results, we extracted six variant dimers (Additional file 1: Fig. S2, Additional file 2: Table S1) with different levels of on-target activity from the screened library. Three of these dimers were selected for their low off-target activity and three further variant pairs were selected with differing levels of off-target activity. To allow rapid retrieval of the selected recombinases, we performed PCR amplification using primers specific for the UMI of the respective clusters (Fig. 2a). We then evaluated the variants using an established plasmid-based assay (Fig. 2b). As reported previously [10], the D7 heterodimer recombined its on-target (loxF8), as well as the previously identified off-target sequences (Fig. 2c, d). The selected recombinase dimers with differing levels of off-target activity (clusters 151, 223, and 435) displayed activity on the on-target and on at least one off-target, with properties similar to the results obtained from the DEQSeq screen (Fig. 2c, d). In contrast, the dimer variants selected for their low off-target activity (clusters 1244, 138, and 181) all recombined loxF8, but showed neglectable recombination on the off-targets, with cluster 1244 being as active as D7, followed by clusters 138 and 181. Therefore, DEQSeq provided reliable results, nominating the recombinase from cluster 1244 as a particularly interesting variant for further investigation.

To test whether the obtained results in *E. coli* translate to human cells, we cloned the three validated variant pairs with high specificity (clusters 138, 181, and 1244) into a mammalian expression vector as monomers and co-transfected the plasmids into HEK293^loxF8^ reporter cells (Fig. 2e, Additional file 1: Fig. S3). As in *E. coli*, the most active recombinase came from cluster 1244, followed by the recombinases obtained from clusters 138 and 181 (Fig. 2f, g). We conclude that DEQSeq identified recombinase variants with valuable properties and improved therapeutic potential.

**Fig. 3:**
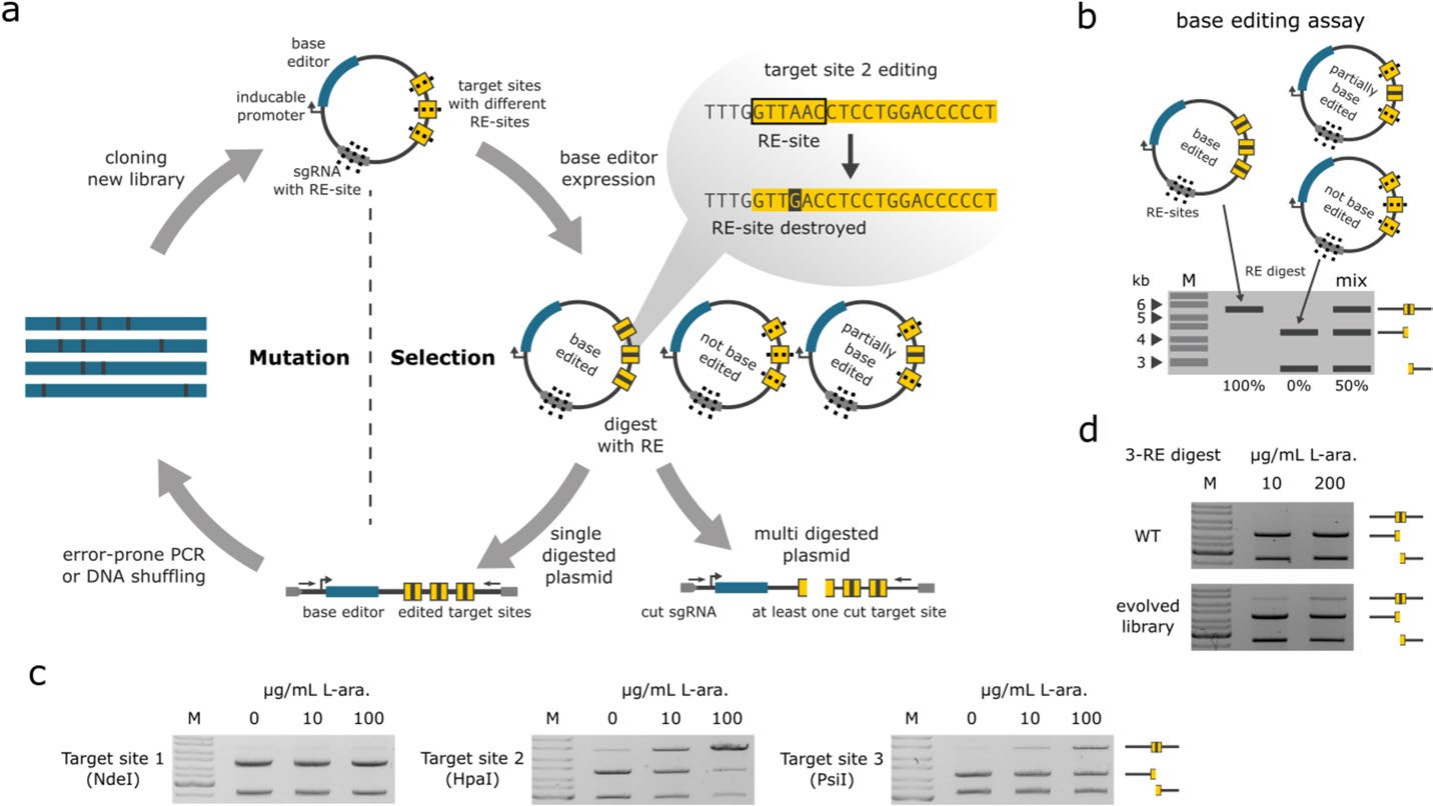
CRISPR-associated substrate-linked directed evolution (CaSLiDE). **a** Schematic illustration of CaSLiDE for base editing. The process starts by cloning a base editor enzyme library (blue) into the pEVO expression vector, which contains expression cassettes for sgRNAs (gray) and sgRNA target sites (yellow). After expression of the base editors, plasmids are isolated and digested with restriction enzymes, which target the sgRNAs and the sgRNA target sites. Applying a restriction digest to the plasmids results in one linear fragment for the edited plasmids and in two fragments for non-edited plasmids. An error-prone PCR using indicated primers (arrows) exclusively generates products of the edited plasmids. These amplified Un1Cas12f1-ABE variants are then subjected to the next evolution cycle. **b** Schematic illustration of the base editing plasmid assay. **c** Plasmid-based assays of the WT Un1Cas12f1-ABE at indicated L-arabinose induction levels. Target sites 1–3 were digested separately by using the corresponding restriction enzymes NdeI, HpaI, or PsiI, respectively. **d** Plasmid-based assay of the evolution starting point and the final evolved library, triple digested simultaneously with NdeI, HpaI, and PsiI. Note the presence of a slower migrating band for the evolved library at indicated L-arabinose induction levels, representing fully edited plasmids

For further evaluation of the DEQSeq approach, we generated Cas12f-ABE variants by adapting the Substrate Linked Directed Evolution (SLiDE, Additional file 1: Fig. S4) [5, 14] technology for the directed evolution of CRISPR-Cas systems (CaSLiDE, Fig. 3a). To accommodate this change, we redesigned the SLiDE plasmid to contain a Un1Cas12f1-TadA8e fusion gene (Cas12f-ABE) driven by a L-arabinose inducible promoter. Additionally, we added an expression cassette for sgRNAs, as well as sequences that are targeted by these sgRNAs. The sgRNA target sites contain restriction enzyme sites, which are altered upon successful base editing, and hence, will not be digested when these restriction enzymes are applied. A subsequent PCR reaction is only successful on non-digested plasmids and will therefore only amplify active base editor sequences. This results in an enrichment of improved base editors over multiple cycles of directed evolution (Fig. 3a). Notably, we included three different sgRNAs and corresponding target sequences on the same plasmid to avoid the emergence of a specific preference for only one target. Using a base editing plasmid assay (Fig. 3b), we found that the original Cas12f-ABE (WT) was active on all three target sites, albeit at low and varying efficiencies (Fig. 3c). We conducted 46 directed evolution cycles and 4 enrichment cycles which resulted in a library of Cas12f-ABE variants with improved editing efficiencies on all three target sites combined (Fig. 3d).

**Fig. 4:**
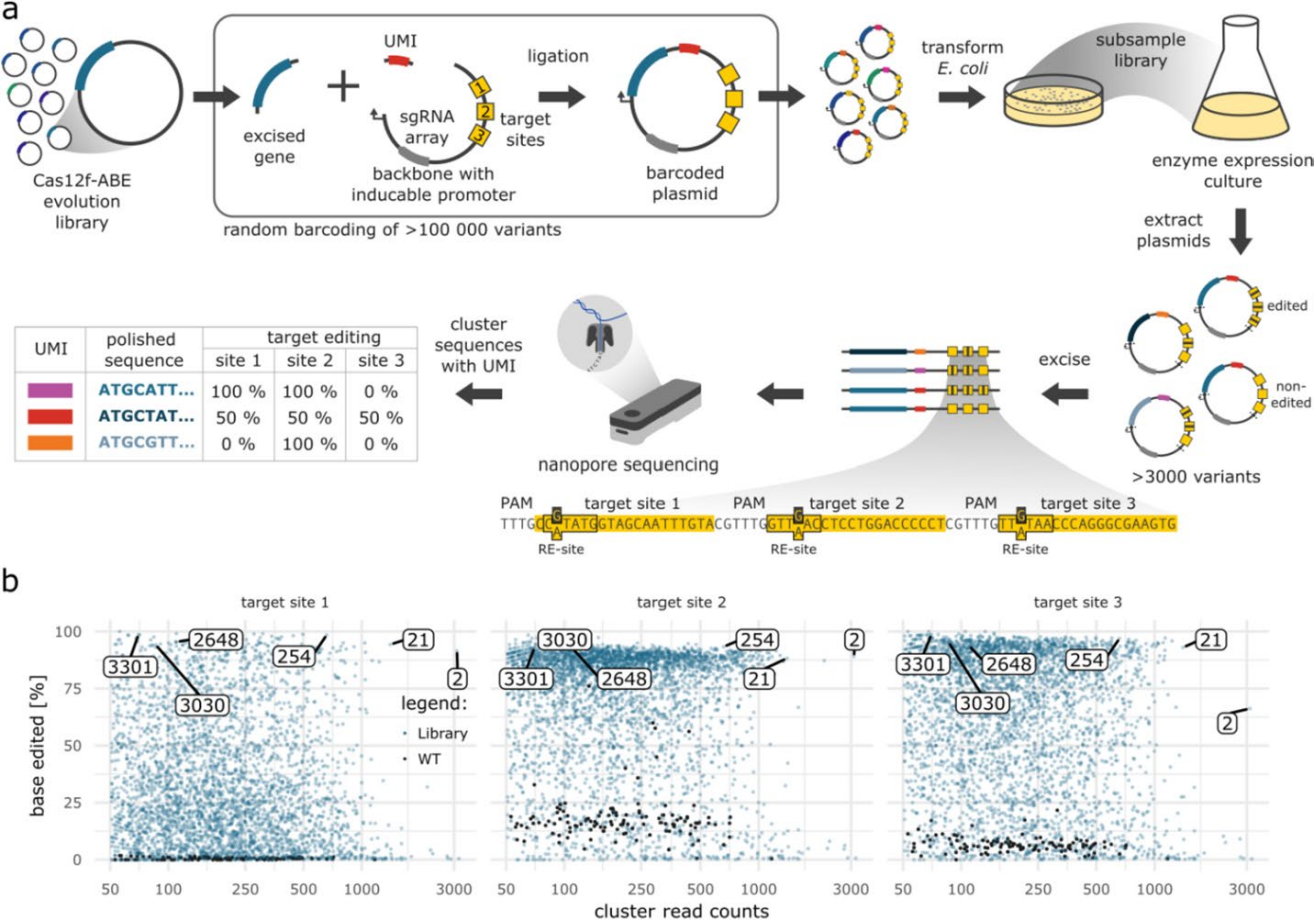
DEQSeq identifies favorable Cas12f-derived miniABEs. **a** Workflow overview. Evolved Cas12f-ABEs are cloned together with a unique molecular identifier (UMI) into a vector containing three target sites (yellow boxes). The number of transformed bacteria is determined by counting colonies on appropriate agar plates. Calculated from the transformation efficiency, a defined number of transformed bacteria is cultured. Cas12f-ABE expression is induced by the addition of L-arabinose, which may result in base editing of the target sites on the plasmid. The plasmid DNA is isolated, and the region containing the Cas12f-ABE gene, the UMI, and the target sites is excised and sequenced with nanopore sequencing. The sequences of the UMIs are computationally clustered based on similarity which results in groups of sequences corresponding to the different variants. From these groups, the base editing events on the different target sites are counted and accurate Cas12f-ABE sequences are generated. **b** DEQSeq screen of Cas12f-ABE on three target sites. UMI-clusters containing evolved Cas12f-ABEs are indicated as blue points and WT control clusters are indicated in black. Clusters that were characterized are highlighted

The evolved Cas12f-ABE library was then screened with DEQSeq (Fig. 4a), where we included the original Un1Cas12f1-ABE8e (WT) as control. In total, the screen yielded 3606 UMI-clusters with 50 or more reads, of which 123 clusters were identified as WT control (Fig. 4b, Additional file 1: Fig. S5a). Analysis of the polished WT sequences revealed only 2 sequence mismatches resulting in a sequencing accuracy of over 99.999%. The determined median editing rates of the WT were 0.5% (target site 1), 16% (target site 2), and 6.2% (target site 3) (Additional file 1: Fig. S5b). Of the 3483 non-WT clusters, 58 had editing rates of more than 90% on all 3 target sites (Additional file 1: Fig. S5c), suggesting that CaSLiDE can be used to rapidly generate base editor variants with improved activity.

**Fig. 5:**
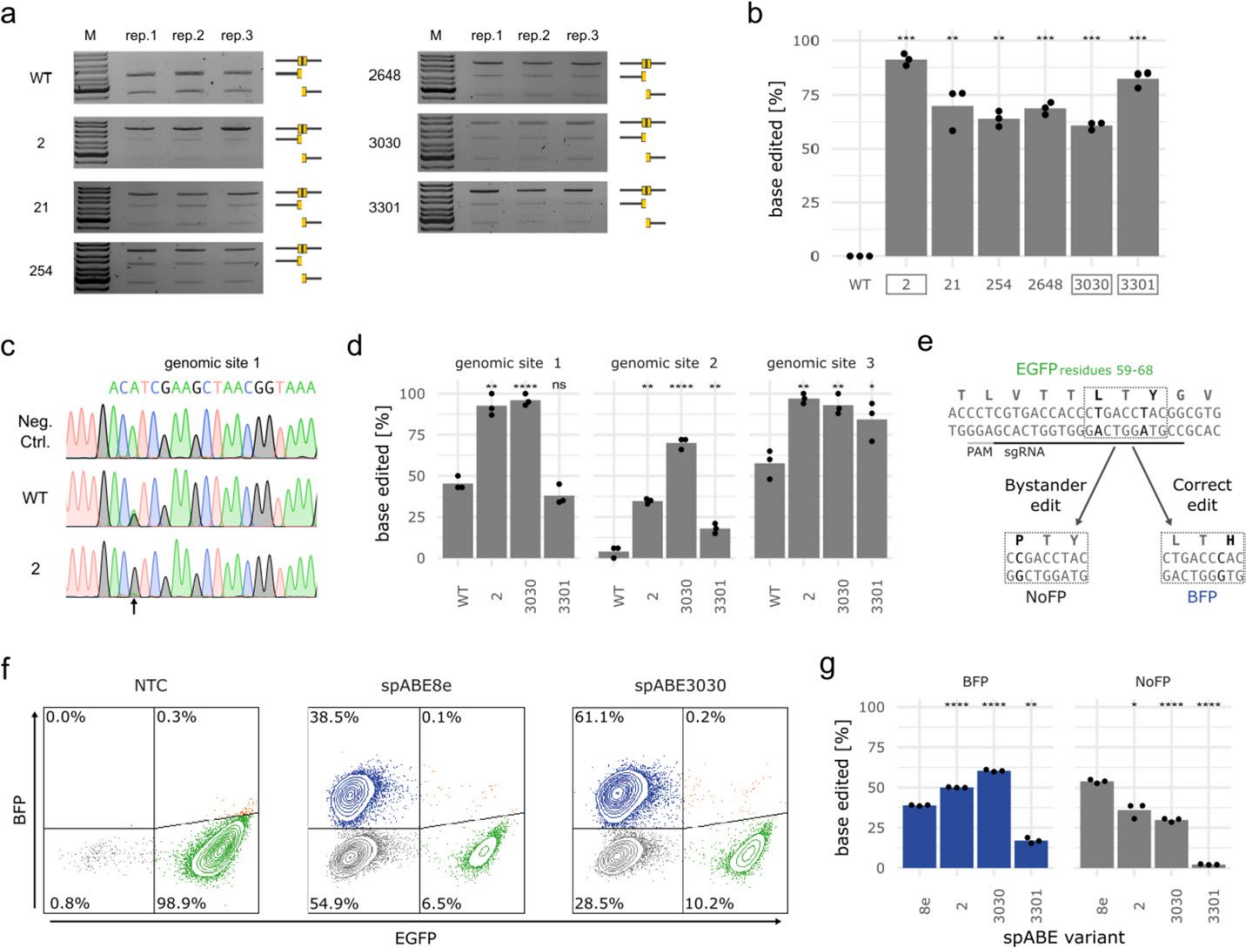
Validation of evolved Cas12f-ABE variants. **a** Agarose gels and **b** quantification of triple target base editing assay of the indicated clusters. The assays were performed with the same induction level as used in the screen. Highlighted with a box are variants that were further validated. Statistical results from t-tests comparing the variants to WT are included above the bars. **c** Sanger sequencing and **d** EditR quantification of *E. coli* gDNA base editing with indicated variants. Statistical results from t-tests comparing the variants to WT are included above the bars. **e** Schematic illustration of the GFP-to-BFP conversion. The employed sgRNA is underlined in black, with the protospacer-adjacent motif (PAM) sequence underlined in gray. The residues Y66 and L64 are marked in black. Bases that can be edited by the spABEs are marked in black. Two editing outcomes are depicted below with the residues and bases changed marked in black. **f** FACS profiles of HEK293T-EGFP reporter cells eight days after nucleofection with indicated ABE mRNAs in combination with the GFP-sgRNA-Y66H. **c** Quantification of GFP to BFP and GFP to no fluorescent protein (NoFP) edited cells. Statistical results from *t*-tests comparing the variants to WT are included above the bars. *P*-values: “ns” not significant, * *p* < 0.05, ** *p* < 0.01, *** *p* < 0.001, **** *p* < 0.0001

To validate these results, we extracted six variants from the Cas12f-ABE library (Additional file 1: Fig. S5b, 6, Additional file 2: Table S1) and evaluated them using a plasmid-based base editing assay (Fig. 3b). The clusters 254, 2648, 3030, and 3301 were chosen for having the highest levels of activity on all target sites and clusters 2 and 21 were chosen for high read counts and high activity values. The assay showed that the variants have editing rates from 60.8 to 91.1%, while for the WT no activity could be detected (Fig. 5a, b and Additional file 1: Fig. S7a). The three variants from clusters 2, 3030, and 3301 were also tested on the genomic DNA of *E. coli*. Three different sgRNAs were tested with the selected variants or the WT control and the editing results were analyzed by Sanger sequencing (Fig. 5c and Additional file 1: Fig. S7b). The quantified base editing rates further validated the superior editing efficiencies of the selected variants over WT (Fig. 5d).

Inspection of the sequences of the isolated clones revealed conserved changes in both the Cas12f, as well as in the TadA coding regions. Because the employed TadA domain had already been optimized by directed evolution [20], we wanted to evaluate whether further improvements of this domain had evolved during CaSLiDE. We therefore fused the ABE8e TadA domain and the TadA domains of clusters 2, 3030, and 3301 with SpCas9 nickase. To benchmark these fusion variants, we produced mRNAs of spABE8e, spABE2, spABE3030, and spABE3301 and transfected equal amounts of mRNAs and sgRNAs into a HEK293T reporter cell line. Successful editing of codon 66 of EGFP in the reporter cell line, turns the protein to BFP and in the case of bystander editing the protein turns nonfunctional (NoFP, Fig. 5e). Using FACS, we observed that spABE2 and spABE3030 showed increased on-target editing when compared to spABE8e, while bystander editing was reduced (Fig. 5f,g). Additionally, we benchmarked spABE3030 to spABE8e at two previously described relevant genomic sites [20] and also evaluated described off-target and bystander editing. In these experiments, we observed similar on-target editing of the two base editors, while spABE3030 showed reduced off-target/bystander editing (Additional file 1: Fig. S7c). These results, although preliminary, indicate that spABE3030 has favorable properties in mammalian cells.

## Discussion

DEQSeq offers remarkable versatility in accommodating a wide array of DNA-editing enzymes for the screening process. In our investigation, we successfully demonstrate the compatibility of the method with both designer-recombinases and Cas12f-ABEs. While our current exploration provides preliminary insights into select variants, a comprehensive characterization demands further attention. Despite the limited scope of variant analysis, the data we present serves to underscore the innovative nature of the DEQSeq approach. By homing in on specific variants, we furnish compelling evidence of its effectiveness. This study, although exploratory, lays a solid foundation, highlighting the potential of DEQSeq as a potent tool for targeted assessments within the realm of DNA editing.

With minor modifications to the SLiDE plasmid used in this screen [5, 14], we anticipate that many types of DNA editing enzymes could be screened with this method. Especially for screening of editors that use Cas9, zinc-finger- or TAL DNA-binding domains, the method should be attractive, as illustrated by the improvement of the TadA domain of the ABE8e base editor. Due to the use of nanopore sequencing, the size of the enzymes is only limited by the maximum possible plasmid size that can be cultured and isolated from bacteria. Additionally, DEQSeq can be used with other plasmid designs and directed evolution strategies, making it very versatile. Therefore, DEQSeq should be amenable to rapidly identify favorable clones from a large number of different DNA-editing enzyme types.

In addition to the high flexibility, DEQSeq has a fast turnaround time, and it is easy to use. The method applies regular cloning approaches and nanopore sequencing to generate the sequencing data. Nanopore sequencing has a low entry cost and allows data to be generated rapidly. Cloning and culture of the barcoded plasmids takes three days and sequencing takes another one to three days. Computational processing of the data requires a capable Linux workstation and basic knowledge of the Linux command-line. Variants of interest can be ordered from gene synthesis companies, or for even faster turnaround time, extracted from the barcoded library using UMI-specific primers. In total, screening of thousands of variants and the extraction of candidate variants can take less than two weeks and the cost and labor required are small in comparison to manual screening approaches.

Besides the methods capacity for identifying noteworthy enzyme variants, DEQSeq serves as a valuable tool for investigating the impact of mutations. The extensive dataset generated by DEQSeq holds the potential to significantly enhance our comprehension of DNA-editing enzymes, facilitating the identification of advantageous mutations aimed at optimizing existing variants. This application can even extend to the realm of employing deep learning techniques to create novel variants, a procedure demanding a substantial volume of sequences to effectively train generative models [21]. Leveraging the rich phenotypic data offered by DEQSeq opens avenues for training models capable of generating enzyme variants characterized by both heightened activity and superior specificity. As we look ahead, forthcoming research endeavors are poised to unveil the true utility of DEQSeq in propelling the evolution of DNA-editing enzymes. The promise of advancing enzyme development through DEQSeq remains a compelling area ripe for exploration and validation.

## Conclusion

DEQSeq offers an efficient high-throughput approach for screening thousands of enzymes simultaneously on multiple target sites with minimal need for resources. With a turnaround time as low as 7 days and only requiring a MinION sequencer and a capable Linux computer, DEQSeq is an economical tool for identifying favorable DNA editing enzymes for research and gene therapy applications. The ability to quickly extract variants of interest from the library through PCR allows for immediate follow-up experiments. Moreover, the data generated provides valuable insights into protein properties and can be used for training of protein generators. Overall, DEQSeq presents a practical and powerful approach for accelerating enzyme discovery and advancing genome editing research.

## Methods

### UMI fragment preparation

To generate a DNA fragment for ligation, a single-stranded DNA oligonucleotide was ordered containing primer binding sites, restriction sites, and 50 random bases. There were two variants of this oligonucleotide depending on which pEVO plasmid it was intended to be used for. For the DEQSeq screen of the evolved Cas12f-ABE variants the “Cas12f UMI fragment” was used while for screen of the loxF8 recombinase variants the “loxF8 UMI fragment” was used (Additional file 3: Table S2). To make these oligonucleotides double stranded a 50 µl PCR was performed with 20 µM of the primers UMIprimer F and UMIprimer R (Additional file 3: Table S2), 10 µM of the oligonucleotide, 10 µl of 5 × MyTaq buffer and 1 µl MyTaq polymerase (Bioline). The PCR-cycler was set 94 °C for 90 s, followed by 10 cycles of 15 s at 94 °C, 15 s at 54 °C and 15 s at 72 °C. The resulting PCR product was digested with SbfI and XbaI for the Cas12f UMI fragment or BsiWI and SbfI for the loxF8 UMI fragment. The digest was then again cleaned up with the Isolate II PCR and Gel Kit (Bioline) and measured with a Qubit HS dsDNA Kit on a Qubit 2.0 (Thermo Fisher Scientific).

### Enzyme variant barcoding

The evolved libraries were acquired from pEVO plasmids by digesting with XbaI and BsrGI-HF for the evolved Cas12f-ABE library or SacI and BsiWI for the loxF8 library. The Cas12f-ABE library and the Cas12f-ABE controls were ligated in a ratio of 60 ng of Cas12f-ABE fragment, 4.8 ng UMI fragment, and 100 ng of BsrGI-HF and SbfI digested pEVO-BE plasmid. The loxF8 library and the D7 controls were ligated in a ratio of 40 ng of the recombinase gene fragment, 1.9 ng of the loxF8 UMI fragment, and 120 ng of SacI and SbfI digested pEVO-loxF8 plasmid.

The ligated plasmids were desalted with MF-Millipore membrane filters (Merck) on distilled water for 30 min and transformed into XL-1 Blue *E. coli* (Agilent) via electroporation. The transformed bacteria were cultured in SOC medium for 30 min at 37 °C. 2 µl of this culture was spread on agarose plates with 15 mg/ml chloramphenicol and incubated over night at 37 °C. The number of colonies on the plates was counted to calculate the number of transformed bacteria present per µl of SOC culture.

To nominate the number of variants for the screen, an amount of the SOC culture equal to the desired number of variants was cultured overnight in 100 ml LB medium with 25 mg/ml chloramphenicol and a defined amount of L-arabinose. For the Cas12f-ABE, around 4000 transformed bacteria per library and 100 transformed bacteria per control were cultured with 10 µg/ml L-arabinose. For the loxF8 recombinase screen, around 4000 transformed bacteria from the library and 50 transformed bacteria of the control were cultured with 1 µg/ml L-arabinose. For each sequencing run, the different libraries and controls were cultured together and the plasmid DNA of these cultures was extracted with the GeneJet Plasmid Miniprep Kit (Thermo Fisher Scientific).

To test the same recombinase variants on multiple target sites, the recombinase DNA from the loxF8 screen was digested with SacI and SbfI and isolated from an agarose gel with the Isolate II PCR and Gel Kit (Bioline). The recombinase dimer fragments were then cloned into pEVO plasmids containing the off-targets of interest. The ligated plasmids were desalted with MF-Millipore membrane filters (Merck) and transformed into XL-1 Blue *E. coli* (Agilent) via electroporation. The transformed bacteria were cultured in SOC medium for 30 min at 37 °C and then transferred to 100 ml LB medium with 25 mg/ml chloramphenicol and 100 µg/ml L-arabinose. The plasmid DNA of these cultures was then extracted with the GeneJet Plasmid Miniprep Kit (Thermo Fisher Scientific).

The barcoded plasmid extracts from the Cas12f-ABE library were digested with ScaI and BsrGI, while the barcoded plasmids from the loxF8 screen were digested with ScaI and SacI. The resulting fragments containing the evolved gene, the UMI, and the target sites were isolated via agarose gel excision with the Isolate II PCR and Gel Kit (Bioline).

### Nanopore sequencing and processing of screen libraries

The DNA concentrations were measured with a Qubit dsDNA HS Assay Kit on a Qubit 2.0 Fluorometer (Thermo Fisher Scientific). Nanopore sequencing library preparation for the loxF8 library was performed according to the “Amplicons by Ligation (SQK-LSK110)” protocol from Oxford Nanopore Technologies, while the Cas12f-ABE library was prepared using “Amplicons by Ligation (SQK-LSK112).” The prepared loxF8 library was then loaded on a MinION FLO-MIN106 flow cell with r9.4.1 pore (Oxford Nanopore Technologies), while the Cas12f library was loaded onto a MinION FLO-MIN110 flow cell with r10.4 pores (Oxford Nanopore Technologies). Sequencing was performed for 72 h. Each screen was performed on one flow cell.

Basecalling of the sequence data was performed on guppy version 6.0.1 with the high accuracy model for the loxF8 library and the super accuracy model for the Cas12f-ABE library (Oxford Nanopore Technologies). Processing of the sequence data was performed on a custom-developed pipeline. Reads were first filtered with Filtlong v0.2.1 to be at least 2900 bp long for the Cas12f-ABE screen and 3000 bp long for the loxF8 screen. Further, Filtlong was also used to filter the reads for a minimum mean Phred quality value of 10 for the loxF8 screen and 18 for the Cas12f-ABE screen. The sequences were then aligned with minimap2 [22] to a reference sequence containing Un1Cas12f1-ABE8e (Cas12f-ABE screen) or D7 (F8 dimer screen) and the UMI consisting of 50 “N.” To ensure coverage of the genes and the UMI the aligned reads were filtered with samtools [23] based on coordinates at the beginning of the enzyme gene and the end of the UMI.

From the filtered alignment, the UMIs were then extracted with the stackStringsFromBam function from the R package GenomicAlignments [24]. The UMIs were then clustered with VSEARCH [25] with a cluster_identity value of 0.7. Sequence reads from clusters with a minimum size of 50 reads were then transferred to separate files and aligned to the gene-UMI reference sequence. These separate read files and alignments were used to construct consensus sequences with racon [26] followed by further polishing with medaka (Oxford Nanopore Technologies), both with standard settings. The polishing process was run in parallel with GNU parallel [27]. Finally, gene sequences were extracted with the R package GenomicAlignments and translated to amino acids.

For the loxF8 screen, the DNA excision rate of the enzymes was calculated by aligning the clustered reads to reference sequences that contain the target site region as a non-recombined and recombined variant. For each target site sequence additional references were provided; this way the different targets could be identified. The recombination rate of the variants was calculated based on the read counts of the target site region alignments.

For the Cas12f-ABE screen, the base editing rate of the enzymes was calculated by aligning the clustered reads to a reference sequence that contains the unedited target site. Using the GenomicAlignments R package, the aligned stacks of 4 bases on the 3 targeted bases were extracted. Editing rates were then determined by counting the correctly edited reads, non-edited reads, and other editing outcome reads.

The results from each screen were then combined and filtered for clusters with 50 reads or more per target site. Control enzymes were identified with the polished variant DNA sequences, those that contain 5 or less mutations in comparison to the control reference sequence were defined as control clusters. All further data processing and visualization was performed in R with the tidyverse [28] and stringdist packages [29]. All computation was performed on a Linux workstation with a 12-core Intel Xeon X5650, 64 GB of memory, and a Nvidia RTX 3600.

### Extraction of enzyme variants

To validate the screens, we extracted enzyme variants from the screened libraries using PCR. We designed reverse primers specific for the UMI of the cluster of interest (Additional file 3: Table S2, “loxF8 UMI-138 R,” “loxF8 UMI-151 R,” “loxF8 UMI-181 R,” “loxF8 UMI-223 R,” “loxF8 UMI-435 R,” “loxF8 UMI-1244 R,” “Cas12f UMI-2 R,” “Cas12f UMI-21 R,” “Cas12f UMI-254 R,” “Cas12f UMI-2648 R,” “Cas12f UMI-3030 R,” “Cas12f UMI-3301 R”) and used them together with a universal forward primer (Additional file 3: Table S2, “loxF8 universal F” for the loxF8 screen and “Evolution F” for the Cas12f-ABE screen) to amplify the enzyme genes we were interested in. The PCRs were performed with a high-fidelity polymerase (Herculase II Fusion DNA Polymerase, Agilent) and digested with XbaI and BsrGI (Cas12f-ABE enzymes) or SacI and BsiWI (loxF8 recombinases) for further cloning.

### Recombination assay

A schematic illustration of the assay is shown in Fig. 2b. pEVO vectors with the different target sites were published previously [10]. Recombinases were cloned into the pEVO vectors with the respective target sites by utilizing SacI and BsiWI restriction enzymes. Expression of recombinases was controlled by an L-arabinose inducible promoter system (araBAD). Recombination of the respective target sites on the evolution plasmid leads to the excision of a 741 bp fragment from the plasmid. The resulting size difference is mediated by the recombinase activity and can be detected by a restriction digest followed by gel electrophoresis. Quantification of the gel images was performed with GelAnalyzer 19.1 (www.gelanalyzer.com).

### pEVO vectors for Un1Cas12f1-ABE8e

To generate a plasmid with the target sites, oligonucleotides containing the target sites (Additional file 3: Table S2) were annealed to form a DNA fragment that was cloned into a BglII digested pEVO backbone via Cold Fusion (System Biosciences). SgRNA (Additional file 4: Table S3) scaffold fragments were synthesized (Twist Bioscience) and cloned into pEVO plasmid containing the target sites utilizing NsiI and NotI restriction enzymes. The pEVO plasmid containing the target sites and the sgRNA scaffold was then used as a template in a PCR reaction where three different sgRNA spacers were included in the reverse primers. In that way, generated sgRNA fragments were then introduced to the pEVO vector in a stepwise manner, by cloning sgRNA1 with NotI and NsiI, sgRNA2 with NsiI, and sgRNA3 via XhoI and SalI. Un1Cas12f1 fragments were produced by Twist Bioscience and amplified via PCR and the TadA gene was prepared via PCR by using the pABE8e-protein plasmid as a template (Addgene plasmid # 161,788). To create Cas12f-ABE, both PCR products were used as a template for an overlap-PCR. The Cas12f-ABE was then cloned into the pEVO-TS-sg1-sg2-sg3 vector with BsrGI and XbaI restriction enzymes. The expression of the Cas12f-ABEs was controlled by the araBAD L-arabinose inducible promoter system.

### Evolution of Cas12f-ABE

A schematic illustration of the procedure can be found in Fig. 3a. First, the Cas12f-ABE library was generated using error-prone-PCR with a low-fidelity DNA polymerase (MyTaq, Bioline and Primers Evolution F and Evolution R (Additional file 3: Table S2) and cloned into the vector with BsrGI and XbaI restriction enzymes (Additional file 1: Fig. S8). After transformation into XL-1 blue *E. coli*, the enzymes were induced at 200 μg/ml L-arabinose. After expression of the base editors, the plasmids were isolated and digested with restriction enzymes, which target the sgRNAs and their target sites. Because the base editing of the target site is happening on the restriction enzyme site, the digest with these enzymes will linearize the edited plasmid, while the non-edited plasmids will be cut into two fragments. This process can be visualized and quantified using agarose gel electrophoresis. The next round of the directed evolution was started with another error-prone-PCR designed to amplify only edited plasmids. The PCR product was then cloned into non-edited pEVO vectors, which started a new evolution cycle. L-arabinose induction and therefore enzyme expression was lowered from 200 μg/ml down to 10 μg/ml during the evolution to increase the selection pressure. Finally, to enrich the library for the DEQSeq screen, four cycles of evolution at 10 μg/ml L-arabinose were performed with a high-fidelity polymerase (Herculase II Fusion DNA Polymerase, Agilent).

### Base editing assay

A Schematic illustration of the assay can be found in Fig. 3b. The assay used the same pEVO plasmid that was used in the directed evolution. The Cas12f-ABE variants were cloned utilizing BsrGI and XbaI restriction enzymes. Expression of the variants was controlled by an L-arabinose inducible promoter system (araBAD). Base editing of the target site results in a loss of a restriction enzyme site. Therefore, restriction digest will linearize the edited plasmid, whereas non-edited plasmids will result in two fragments, which can be detected with gel electrophoresis. Quantification of the gel images was performed with GelAnalyzer 19.1 (www.gelanalyzer.com).

#### Modification of the *E. coli* genome

Three different sgRNA (Additional file 4: Table S3, “sgE_Coli1,” “sgE_Coli2,” “sgE_Coli3”) were cloned into the pEVO plasmid, which target genomic DNA of *E. coli*. Cas12f-ABE WT and 3 variants (clusters 2, 3030, 3301) were cloned into these vectors utilizing BsrGI and XbaI restriction enzymes and transformed into *E. coli* cells. After an overnight culture in LB-medium with 1 µg/ml L-arabinose the cells were spun down and resuspended in 200 µl ddH2O. This suspension was then heated to 95 °C for 10 min and spun down again. The supernatant, which contained the gDNA was then used for a PCR reaction that produces a DNA fragment containing all three sgRNA target sites (Primers “E_Coli gDNA F” and “E_Coli gDNA R,” Additional file 3: Table S2). The PCR products were sequenced via Sanger sequencing and the editing rates were analyzed with EditR [30].

#### In vitro mRNA transcription

To generate a base editing plasmid for eukaryotic cells, ABE8e was amplified via overlap-PCR by using the pABE8e-protein plasmid as template (Addgene plasmid # 161,788; Additional file 3: Table S2, primers “ABE8e F,” “Linker mod R,” “Linker mod F,” and “ABE8e R”) and cloned into pLenti-mCherry plasmid with NotI and XbaI restriction enzymes. Additionally, we included a linker with the unique restriction site BspEI between TadA and SpCas9. TadA domains of clusters 2, 3030 and 3301 (primer “TadA F,” “TadA c2 R,” “TadA c3030 R,” and “TadA c3301 R”) were amplified via PCR using the respective pEVO plasmids as template and were cloned into pLenti-ABE8e-mCherry with NotI and BspEI restriction enzymes. The PCRs were performed with a high-fidelity polymerase (Herculase II Fusion DNA Polymerase, Agilent). In vitro transcribed (IVT) mRNA was prepared from a PCR amplicon carrying the gene of interest, which were generated with the primers ABE mRNA F and ABE mRNA R (Additional file 3: Table S2) and Herculase II Fusion DNA Polymerase (Agilent, Santa Clara, CA, USA). IVT mRNAs were generated according to the manufacturer’s guidelines using the HiScribe T7 ARCA mRNA Kit (NEB, Ipswich, MA, USA) followed by purification using Monarch RNA Cleanup Kit (NEB, Ipswich, MA, USA).

### Cell culture of HEK293T cells

HEK293T (ATCC) were cultured in DMEM, Dulbecco’s modified Eagle’s medium (Gibco) with 10% fetal bovine serum and 1% penicillin–streptomycin (10,000 U/ml, Thermo Fisher). The cells were incubated, maintained, and cultured at 37 °C with 5% CO_2_. The cell line was authenticated by the supplier and tested negative for mycoplasma.

### HEK293T^loxF8^ reporter cells transfection with plasmids

Each recombinase monomer of the dimmers was cloned in the transient mammalian expression vector (EF1a-Rec1-P2A-EGFP) or (EF1a-Rec2-P2A-tagBFP). One day before transfection 2 × 10^5^ cells were plated in 24-well format to reach 80% confluency at the time of transfection. Each monomer was co-transfected (0.75 µg each plasmid) with 2 µl Lipofectamine 2000 (Thermo Fisher) reagent. Recombination was measured via FACS 72 h after transfection.

### HEK293T cells nucleofection with mRNA

For mRNA nucleofection 2 × 10^5^ HEK293T-EGFP cells were resuspended with 20 µL supplemented nucleofector solution from SF Cell Line 4D-Nucleofector™ X Kit S (Lonza) with 1 pmol ABE mRNAs and 10 pmol sgRNAs (Synthego) and nucleofected with 4D-Nucleofector Core and X Unit (Lonza, Basel, Switzerland), program CM-130.

### Genomic DNA isolation

Genomic DNA was isolated 72 h post-transfection using the QIAamp DNA Blood Mini Kit (Qiagen). 250 ng of gDNA was used for a PCR reaction using Q5 High-Fidelity DNA Polymerase (New England Biolabs). The PCR fragments were sequenced by Sanger sequencing.

### Fluorescent-activated cell analysis

HEK293T cells were washed once with PBS and then detached using Trypsin (Gibco) cells were resuspended in DMEM and analyzed with the at BD™ LSR Fortessa (BD Biosciences).

### Supplementary Information

**Additional file 1:** **Figure S1. **Analysis of single variants from the designer-recombinase screen. (a)DEQSeq results shown as recombination percentages of the 53 identified D7 controls on the indicated target sites. The box plots are according to standard definition: median for the center line, upper and lower quartiles for the box limits, 1.5x interquartile range for the whiskers. The single values are shown as grey points. (b)Screen results of indicated clusters with more than 25% recombination on loxF8 (dark blue) and less than 10% recombination on the three off-targets (light blue, green and yellow). **Figure S2. **Amino acid sequence alignments of indicated recombinases. Sequences of the left recombinase monomers are shown in (a),whereas the right recombinase monomers are shown in (b). A dot indicates conservation to D7. **Figure S3. **Flow cytometry gating strategies to evaluate recombination efficiencies. (a)Gating strategy used for the analysis of the basal levels of mCherry expression in the reporter cell line HEKloxF8. (b)Gating strategy used for the analysis of the recombination efficiency.**Figure S4. **Overview of the substrate-linked directed evolution (SLiDE) workflow. SLiDE starts by cloning recombinase libraries (blue) into the pEVO expression vector, which contains two lox-like sites (yellow triangles). After expression of the recombinases, plasmids are isolated and digested with restriction enzymes present between the lox-like sites. Applying a restriction digest will linearize the nonrecombined plasmids, while recombined plasmids remain circular. An error-prone-PCR (primers indicated as arrows) will exclusively generate a product from recombined plasmids. Sequences can also be diversified by DNA shuffling. The amplified and mutated active recombinase variants are then subjected to the next evolution cycle. **Figure S5. **Analysis of single variants from the Cas12f-ABE screen. (a) DEQSeq results shown as percentages of edited reads from the WT controls on the three target sites. The box plots are according to standard definition: median for the center line, upper and lower quartiles for the box limits, 1.5x interquartile range for the whiskers. The single values are shown as grey points. (b)DEQSeq base editing outcomes of the selected variants based on the sequence that matches the positions two to five on the target site. The expected edit is supposed to happen on an adenine at position 3 or 4. Blue shows the percentage of correctly edited reads, light grey shows the percentage of reads where no editing happened and dark grey shows the percentages of all other editing outcome reads. (c)Screen results of clusters with over 90% base editing on the indicated target sites are shown. **Figure S6. **Amino acid sequence alignment of the indicated Cas12f-ABE variants to WT. A dot indicates conservation to WT. Protein regions are indicated with a colored bar on top of the sequence alignment. **Figure S7. **Separate validations of the selected Cas12f-ABEs. (a) Plasmid-based quantification of base editing of Cas12f-ABE WT and the indicated variants. Percentages of base editing by single digest with the NdeI, HpaI or PsiI restriction enzymes. (b)Representative Sanger sequencing chromatograms of base edited *E. coli *gDNA. Correctly positioned “A” peaks (blue) are converted into “G” peaks (black) by the base editor. Editing positions are indicated by an arrow. Note the only synonymous bystander edit at position 12 “A” with sgRNA2 is indicated by a blue arrow. The schematics on top show the sgRNA targeted genomic sequence (20 bp). (c)Base editing of spCas9 linked to ABE8e and the evolved ABE3030 at the genomic sites VEGFA3, EMX1 and their off-targets/bystander edits. All A-to-G conversions within each protospacer are shown. **Figure S8. **Plasmid map of a pEVO containing the WT Un1Cas12f1-ABE, the guide RNA array and the corresponding three target sites. Plasmid map was generated using SnapGene 7.0.

**Additional file 2.** Protein sequences of analyzed variants.

**Additional file 3.** List of oligos used in this study.

**Additional file 4.** List of sgRNAs used in this study.

**Additional file 5.** Review history.

## Data Availability

The sequence data generated in this study are available at the European Nucleotide Archive under the accession number PRJEB67459 [31]. The most up-to-date code for DEQSeq is available at https://github.com/ltschmitt/DEQSeq [32] and the version that was used in this study can be found at https://doi.org/10.5281/zenodo.8298510 [33].
