# Supplementary material for: Quantification of evolved DNA-editing enzymes at scale with DEQSeq
Source: Genome Biol. 2023 Nov 6;24:254. doi: 10.1186/s13059-023-03097-3 (PMC10626641; doi:10.1186/s13059-023-03097-3)
Supplement: Supplementary file 1 — Additional file 1: Figure S1. Analysis of single variants from the designer-recombinase screen. (a)DEQSeq results shown as recombination percentages of the 53 identified D7 controls on the indicated target sites. The box plots are according to standard definition: median for the center line, upper and lower quartiles for the box limits, 1.5x interquartile range for the whiskers. The single values are shown as grey points. (b)Screen results of indicated clusters with more than 25% recombination on loxF8 (dark blue) and less than 10% recombination on the three off-targets (light blue, green and yellow). Figure S2. Amino acid sequence alignments of indicated recombinases. Sequences of the left recombinase monomers are shown in (a),whereas the right recombinase monomers are shown in (b). A dot indicates conservation to D7. Figure S3. Flow cytometry gating strategies to evaluate recombination efficiencies. (a)Gating strategy used for the analysis of the basal levels of mCherry expression in the reporter cell line HEKloxF8. (b)Gating strategy used for the analysis of the recombination efficiency.Figure S4. Overview of the substrate-linked directed evolution (SLiDE) workflow. SLiDE starts by cloning recombinase libraries (blue) into the pEVO expression vector, which contains two lox-like sites (yellow triangles). After expression of the recombinases, plasmids are isolated and digested with restriction enzymes present between the lox-like sites. Applying a restriction digest will linearize the nonrecombined plasmids, while recombined plasmids remain circular. An error-prone-PCR (primers indicated as arrows) will exclusively generate a product from recombined plasmids. Sequences can also be diversified by DNA shuffling. The amplified and mutated active recombinase variants are then subjected to the next evolution cycle. Figure S5. Analysis of single variants from the Cas12f-ABE screen. (a) DEQSeq results shown as percentages of edited reads from the WT controls on the three [file 13059_2023_3097_MOESM1_ESM.pdf]

Quantification of evolved DNA-editing enzymes at scale with DEQSeq

Lukas Theo Schmitt[1,2], Aksana Schneider[1,2], Jonas Posorski[1], Felix Lansing[1,2], Milica Jelicic[1], Manavi Jain[1], Shady Sayed[1], Frank Buchholz[1,*] and Duran Sürün[1,*]

[1]Medical Faculty and University Hospital Carl Gustav Carus, UCC Section Medical Systems Biology, TU Dresden, 01307 Dresden, Germany

[2]Current address: Seamless Therapeutics GmbH, Tatzberg 47/49, 01307 Dresden, Germany

*Correspondence should be addressed to F.B. (frank.buchholz@tu-dresden.de) or D.S. (duran.sueruen@tu-dresden.de).

Supplementary Figures

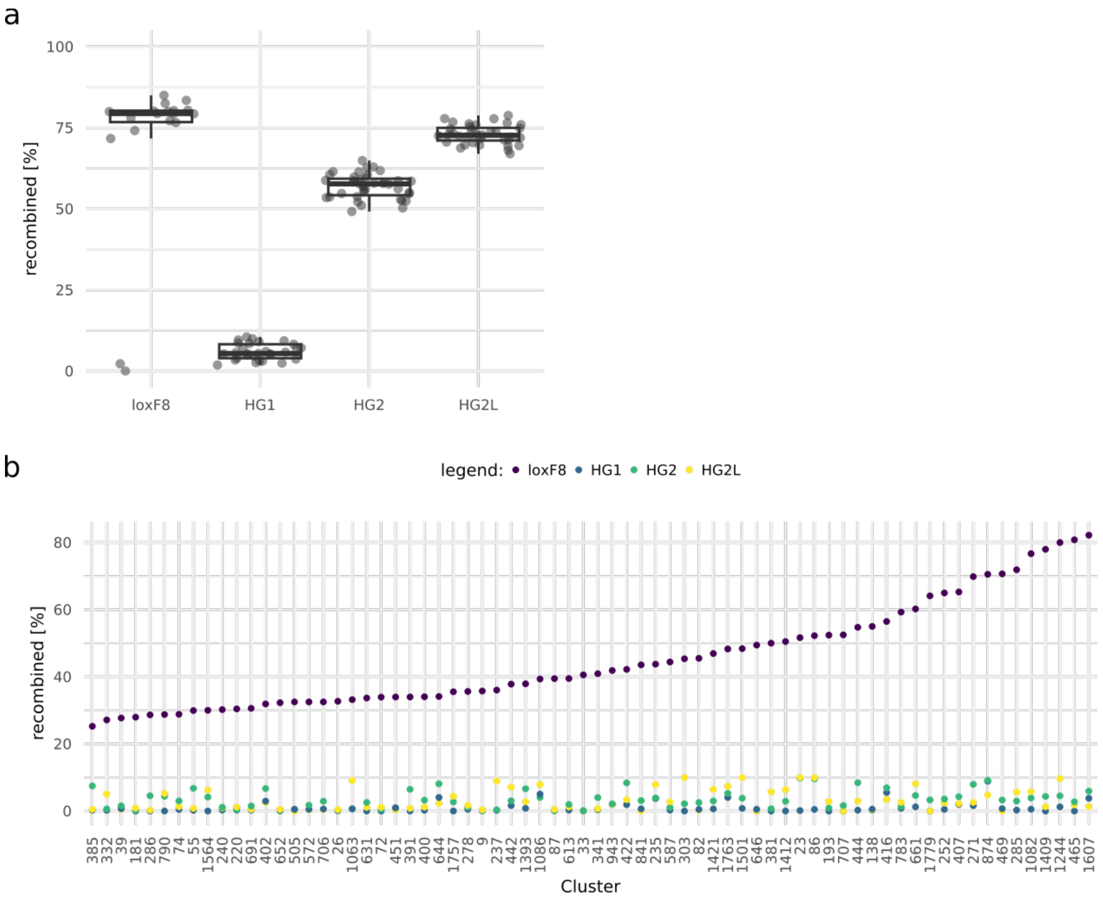

Figure S1: Analysis of single variants from the designer-recombinase screen. **(a)** DEQSeq results shown as recombination percentages of the 53 identified D7 controls on the indicated target sites. The box plots are according to standard definition: median for the center line, upper and lower quartiles for the box limits, 1.5x interquartile range for the whiskers. The single values are shown as grey points. **(b)** Screen results of indicated clusters with more than 25% recombination on loxF8 (dark blue) and less than 10% recombination on the three off-targets (light blue, green and yellow).

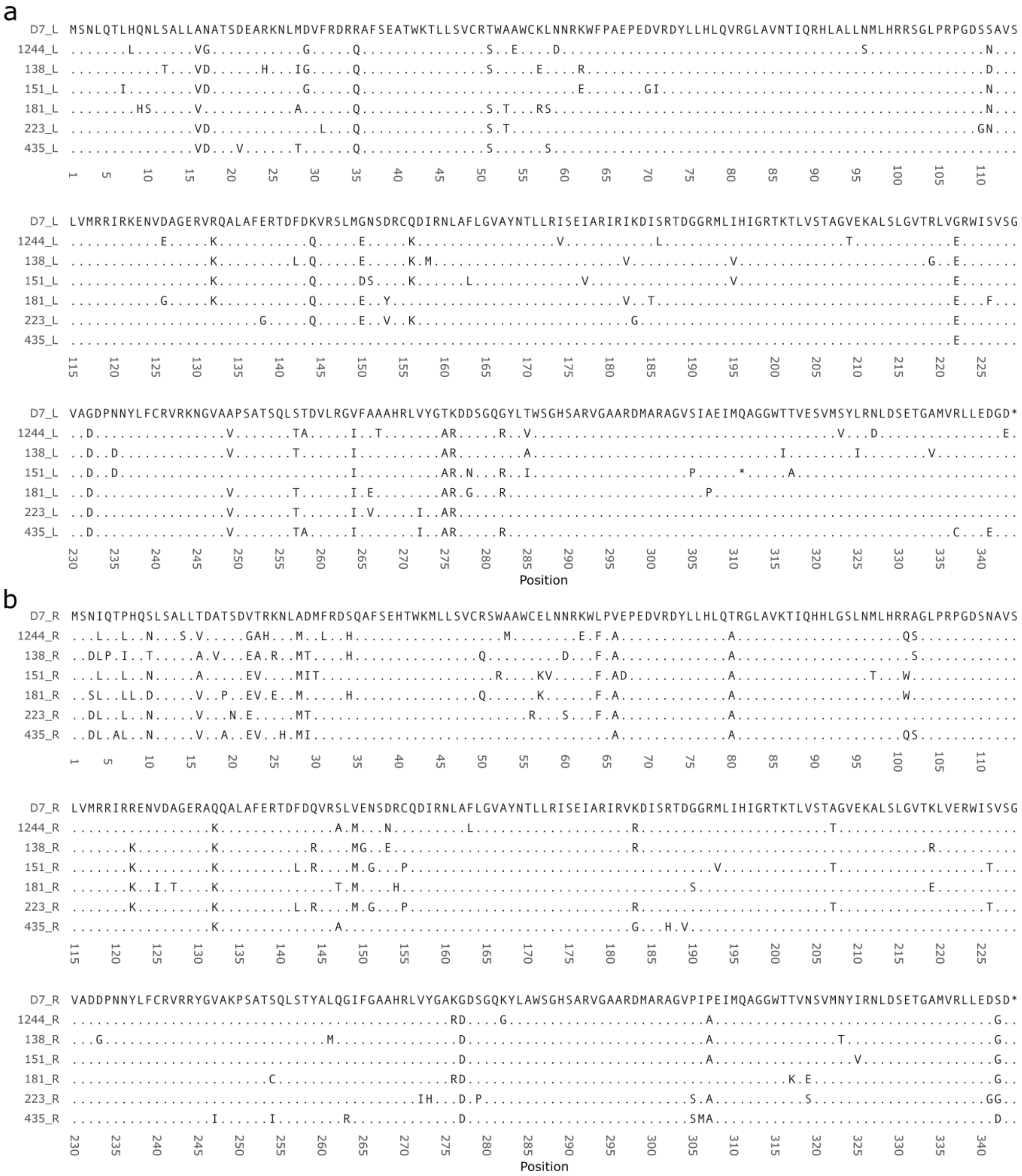

Figure S2: Amino acid sequence alignments of indicated recombinases. Sequences of the left recombinase monomers are shown in **(a),** whereas the right recombinase monomers are shown in **(b)**. A dot indicates conservation to D7.

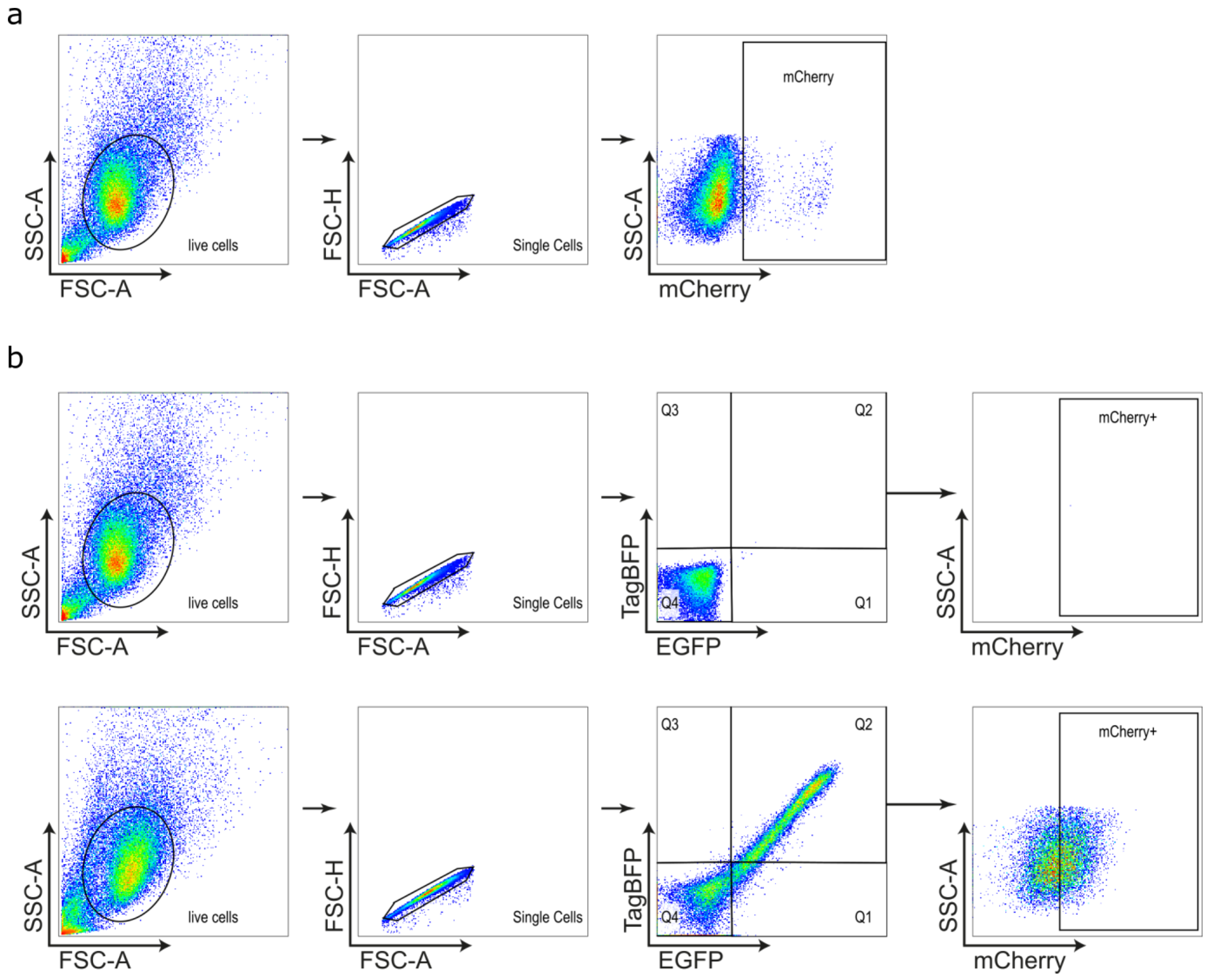

Figure S3: Flow cytometry gating strategies to evaluate recombination efficiencies. **(a)** Gating strategy used for the analysis of the basal levels of mCherry expression in the reporter cell line HEK^{loxF8}. **(b)** Gating strategy used for the analysis of the recombination efficiency.

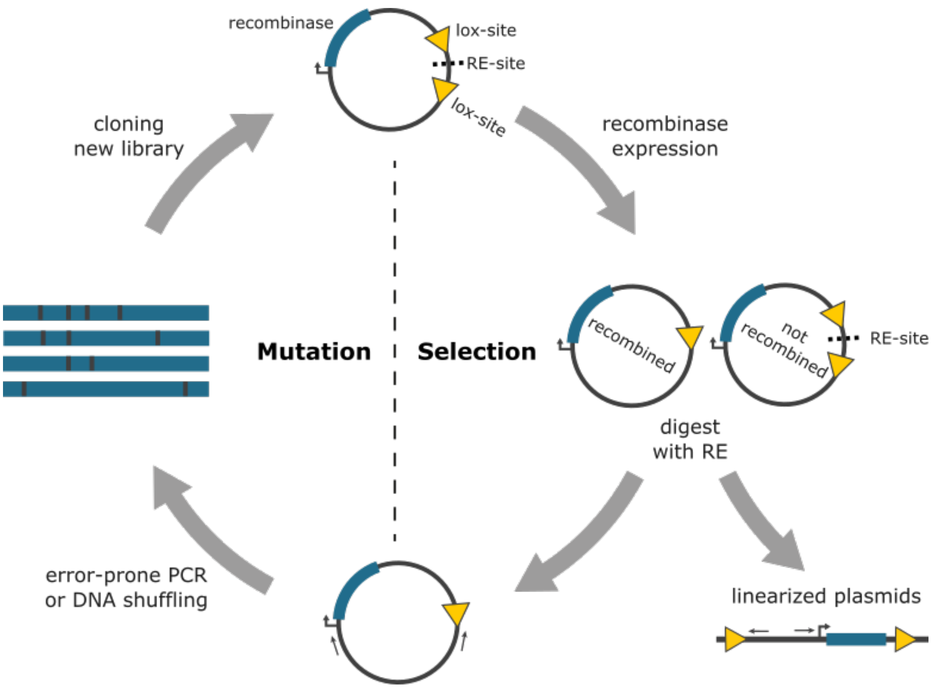

Figure S4: Overview of the substrate-linked directed evolution (SLiDE) workflow. SLiDE starts by cloning recombinase libraries (blue) into the pEVO expression vector, which contains two lox-like sites (yellow triangles). After expression of the recombinases, plasmids are isolated and digested with restriction enzymes present between the lox-like sites. Applying a restriction digest will linearize the non-recombined plasmids, while recombined plasmids remain circular. An error-prone-PCR (primers indicated as arrows) will exclusively generate a product from recombined plasmids. Sequences can also be diversified by DNA shuffling. The amplified and mutated active recombinase variants are then subjected to the next evolution cycle.

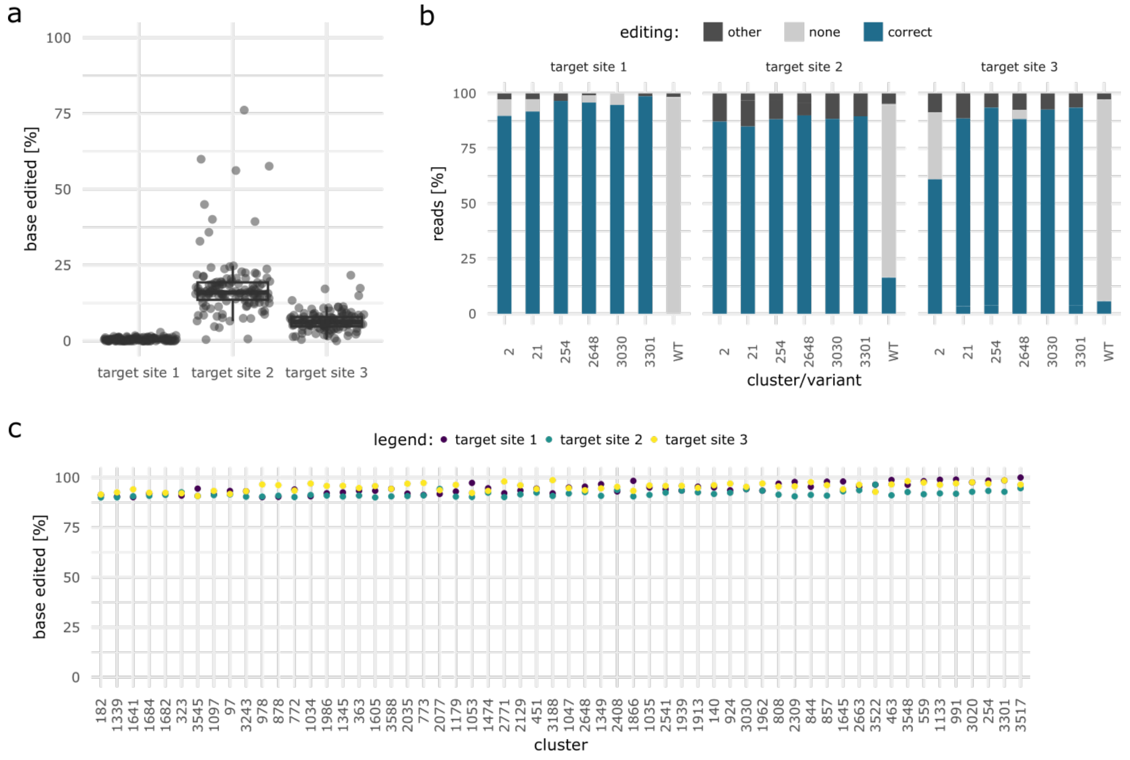

Figure S5: Analysis of single variants from the Cas12f-ABE screen. **(a)** DEQSeq results shown as percentages of edited reads from the WT controls on the three target sites. The box plots are according to standard definition: median for the center line, upper and lower quartiles for the box limits, 1.5x interquartile range for the whiskers. The single values are shown as grey points. **(b)** DEQSeq base editing outcomes of the selected variants based on the sequence that matches the positions two to five on the target site. The expected edit is supposed to happen on an adenine at position 3 or 4. Blue shows the percentage of correctly edited reads, light grey shows the percentage of reads where no editing happened and dark grey shows the percentages of all other editing outcome reads. **(c)** Screen results of clusters with over 90% base editing on the indicated target sites are shown.

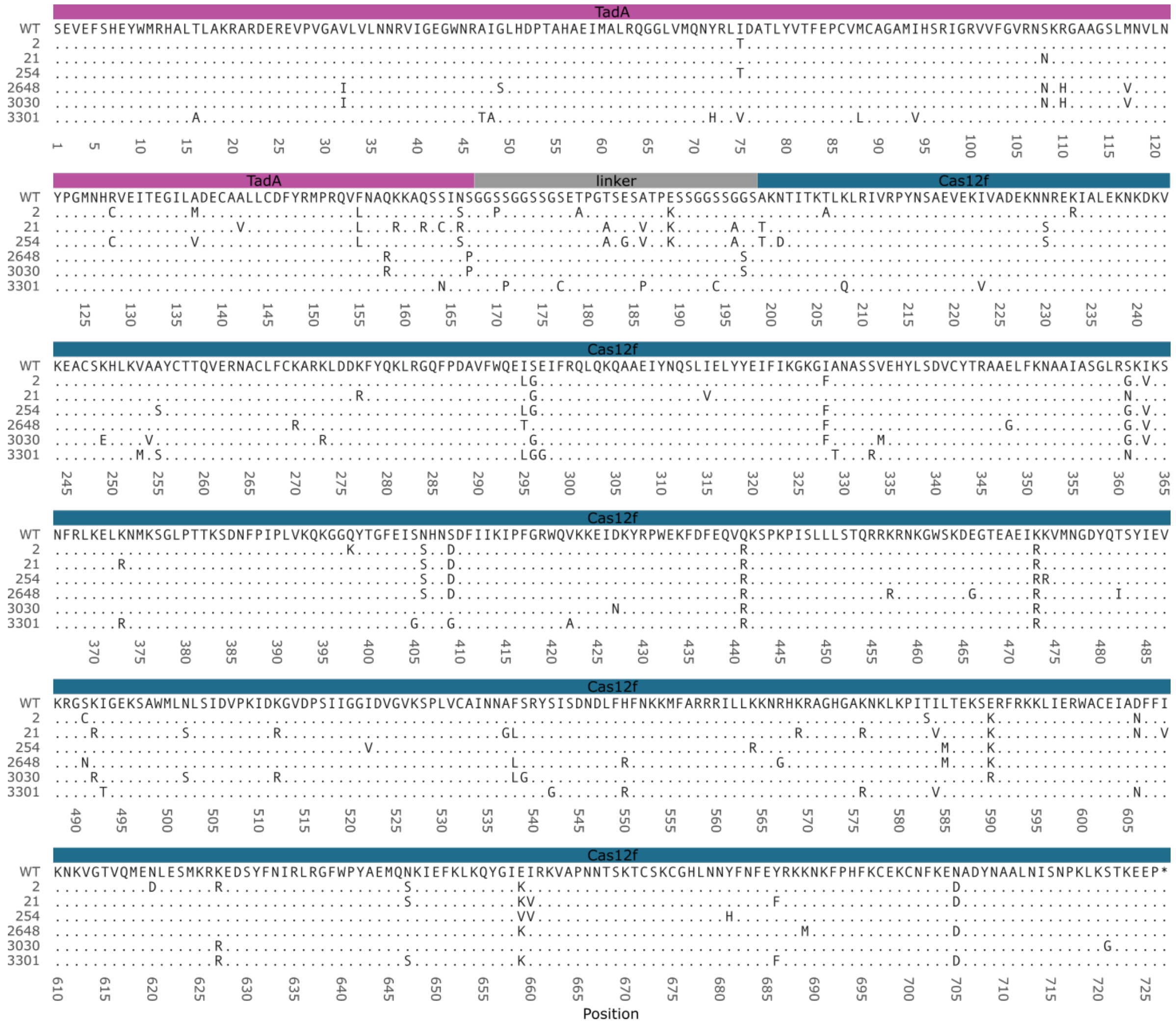

Figure S6: Amino acid sequence alignment of the indicated Cas12f-ABE variants to WT. A dot indicates conservation to WT. Protein regions are indicated with a colored bar on top of the sequence alignment.

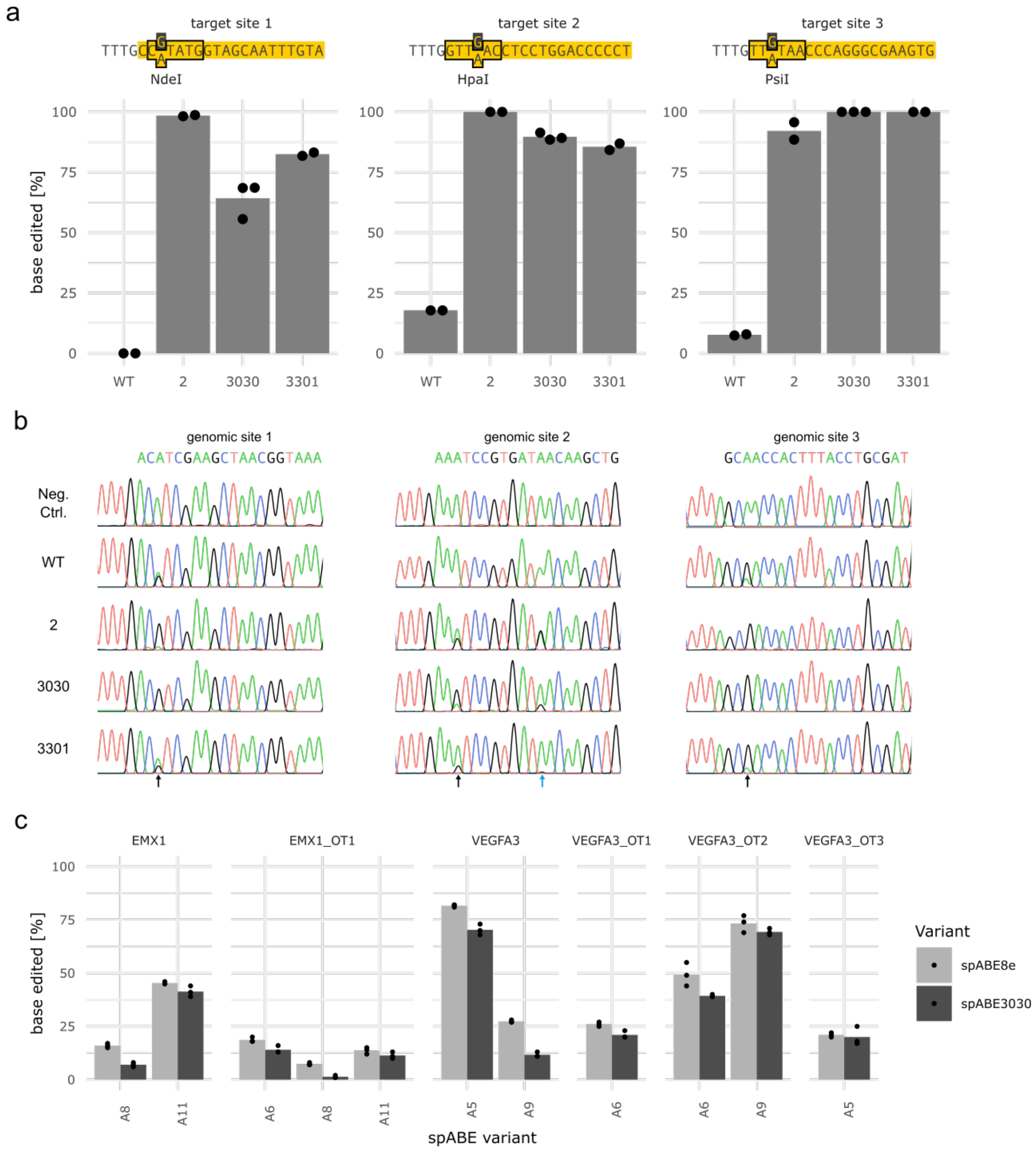

Figure S7: Separate validations of the selected Cas12f-ABEs. **(a)** Plasmid-based quantification of base editing of Cas12f-ABE WT and the indicated variants. Percentages of base editing by single digest with the NdeI, HpaI or PsiI restriction enzymes. **(b)** Representative Sanger sequencing chromatograms of base edited *E. coli* gDNA. Correctly positioned “A” peaks (blue) are converted into “G” peaks (black) by the base editor. Editing positions are indicated by an arrow. Note the only synonymous bystander edit at position 12 “A” with sgRNA2 is indicated by a blue arrow. The schematics on top show the sgRNA targeted genomic sequence (20 bp). **(c)** Base editing of spCas9 linked to ABE8e and the evolved ABE3030 at the genomic sites VEGFA3, EMX1 and their off-targets/bystander edits. All A-to-G conversions within each protospacer are shown.

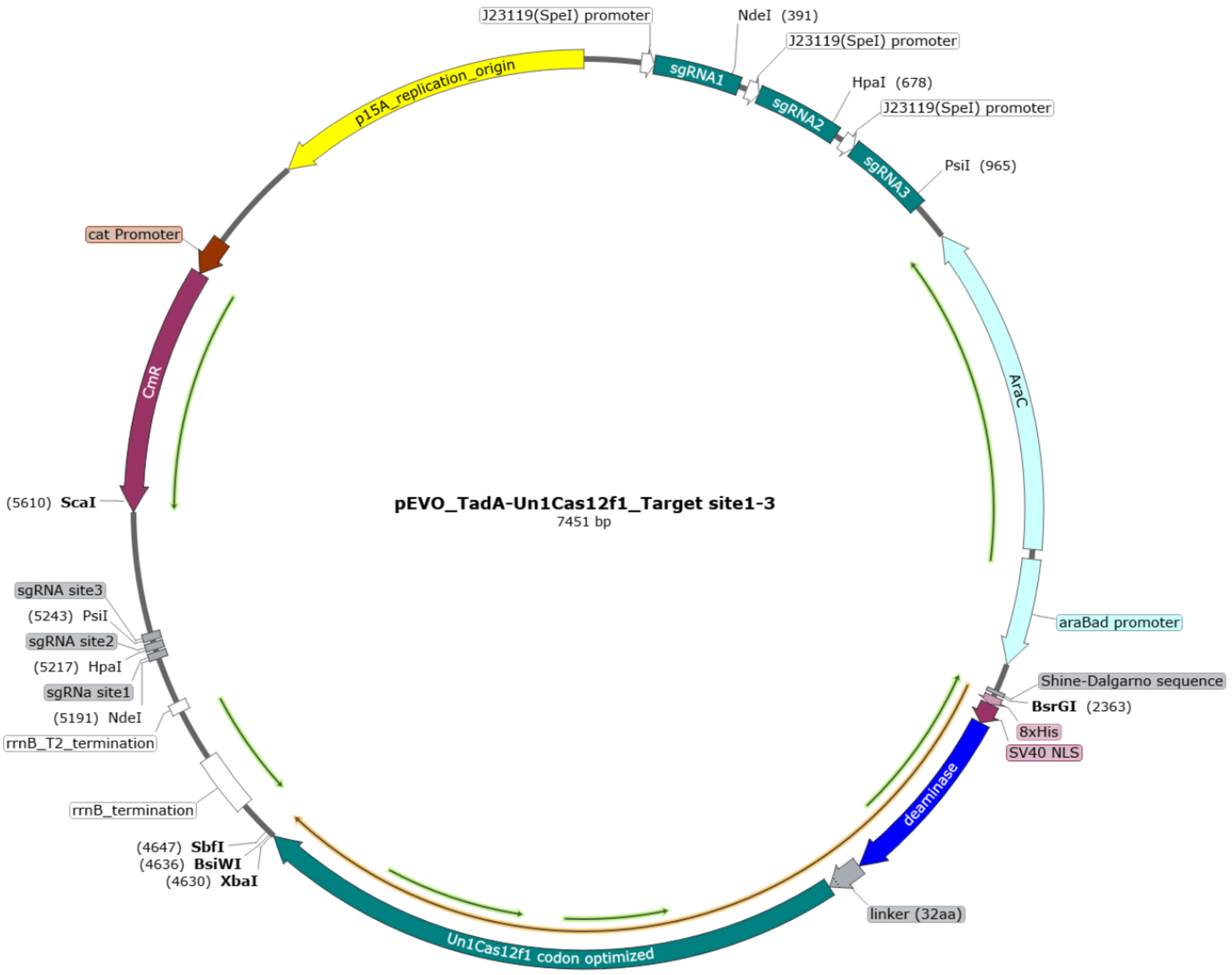

Figure S8: Plasmid map of a pEVO containing the WT Un1Cas12f1-ABE, the guide RNA array and the corresponding three target sites. Plasmid map was generated using SnapGene 7.0.
