# Supplementary material for: Quantification of evolved DNA-editing enzymes at scale with DEQSeq
Source: Genome Biol. 2023 Nov 6;24:254. doi: 10.1186/s13059-023-03097-3 (PMC10626641; doi:10.1186/s13059-023-03097-3)
Supplement: Supplementary file 5 — Additional file 5. Review history. [file 13059_2023_3097_MOESM5_ESM.docx]

Review History

**First round of review**

**Reviewer 1**

**Were you able to assess all statistics in the manuscript, including the appropriateness of statistical tests used?** No, I do not feel adequately qualified to assess the statistics.

**Were you able to directly test the methods?** No.

**Comments to author:**

In this manuscript, the authors present a high-throughput screening platform combining UMI clustering and nanopore sequencing to characterize thousands of DNA editing enzyme variants on multiple target sites, and demonstrated the applicability of this platform on two different DNA editing systems. A few of my concerns for the authors to consider:

1. I am not very convinced by the statement "to our knowledge none of these enable the efficient linkage of genotype and phenotype information retrieval at scale". I hope that the authors can further elaborate and be cautious of using such a firm statement. As protein directed evolution is based on phenotypic outcome as a screening criterion, functional variants are selected from a library of various genotypes.

2. 3-candidate variants (138, 181, 1244 for recombinase variants, and 2, 3030, 3301 for Cas12f-ABE) were selected to validate the DEQSeq result. However, the manuscript did not provide a clear explanation on which specific variant was optimal and how the authors determined its importance. Based on the information provided, "optimal" in the sentence "…selection of optimal genome enzymes from directed molecular evolution experiments…" may not accurate.

3. The manuscript does not provide information comparing off-target activity of evolved high editing efficiency Cas12f-ABE variants with Un1Cas12f1-ABE8e across the whole genome. If the off-target results demonstrate comparable levels of activity, it would serve as a strong endorsement for the effectiveness of DEQSeq.

4. Could the authors consider inserting an enzyme cleavage site between the two loxF8 site and digesting with this enzyme prior to subcloning the 3 other target backbones (HG1, HG2, HG2L)? This could potentially help exclude variants that are not recombinantly active on the loxF8 site.

Minor points:

1. Please add statistical methods in Fig. 1e and Fig. 2e.

2. In Fig.1 and 2, before transformed to E. coli, the plasmid library diagram missed the loxF8 site and promoter, target site of 3 sgRNA and sgRNA.

3. The format of reference 15 should remain consistent with others.

**Reviewer 2**

**Were you able to assess all statistics in the manuscript, including the appropriateness of statistical tests used?** No, I do not feel adequately qualified to assess the statistics.

**Were you able to directly test the methods?** No.

**Comments to author:**

This is a very interesting study that reports a widely applicable method to evolve and engineer DNA recombination and engineering enzymes. The authors smartly combine the enzyme-coding sequence variance and the target in sequence so that they can be sequenced together with long-read nanopore sequencing. Their platform was demonstrated with engineering designer recombinases and Un1Cas12f1-ABE systems.

Major points:

1) The manuscript is too succinct to follow. The rationale and design are hidden in the Figure legends and Methods. This is not the best way to write a paper. Based on the content, it would be nicer to write it as a full-length research article.

2) In both cases (designer recombinase and Un1Cas12f1-ABE), what's the rationale for picking the three variants each? Was that truly random? It would be valuable to pick more clones for validation so that we can learn how reliable the system is and what kind of mutations are behind the improved activity.

3) The authors listed protein alignments of the three highly efficient Un1Cas12f-ABE systems. Please provide full protein sequences and annotated vector sequences for readers to extract such information. Also, I suggest depositing these vectors to Addgene because many would be interested in testing and applying these improved ABEs in their organisms of interest.

4) Will the system work well with larger enzymes and hence longer coding sequences? What will be the challenges in this case? Some interesting discussion on this point and others would make this paper more interesting.

5) Will the evolved work well in eukaryotic cells? Validation on this will add much to this story.

Minor point:

1) Page 5 line 64-69: Please mention the 1244 clone as this one showed reduced off-target effects without compromising the on-target recombination activity.

**Authors Response**

**Point-by-point responses to the reviewers’ comments:**

Reviewer #1: In this manuscript, the authors present a high-throughput screening platform combining UMI clustering and nanopore sequencing to characterize thousands of DNA editing enzyme variants on multiple target sites, and demonstrated the applicability of this platform on two different DNA editing systems. A few of my concerns for the authors to consider:

**Reply: We thank reviewer#1 for the comments and suggestions that we have addressed as outlined below.**

1.      I am not very convinced by the statement "to our knowledge none of these enable the efficient linkage of genotype and phenotype information retrieval at scale". I hope that the authors can further elaborate and be cautious of using such a firm statement. As protein directed evolution is based on phenotypic outcome as a screening criterion, functional variants are selected from a library of various genotypes.

**Reply: We thank the reviewer for this cautious note. We have toned down the statement. We have also extended the background section and more specifically elaborate on limitations of other methods.**

2.      3-candidate variants (138, 181, 1244 for recombinase variants, and 2, 3030, 3301 for Cas12f-ABE) were selected to validate the DEQSeq result. However, the manuscript did not provide a clear explanation on which specific variant was optimal and how the authors determined its importance. Based on the information provided, "optimal" in the sentence "…selection of optimal genome enzymes from directed molecular evolution experiments…" may not accurate.

**Reply: We agree with the reviewer that “optimal” could be misleading in this context. We have therefore changed the wording to “favorable”. In addition, we have extended the validation to 6-candidate variants for the recombinases and the ABE.**

3.      The manuscript does not provide information comparing off-target activity of evolved high editing efficiency Cas12f-ABE variants with Un1Cas12f1-ABE8e across the whole genome. If the off-target results demonstrate comparable levels of activity, it would serve as a strong endorsement for the effectiveness of DEQSeq.

**Reply: We acknowledge the importance of thoroughly characterizing the selected variants, but it's crucial to note that a comprehensive analysis of the variants goes beyond the scope of this manuscript. The focus of the current work lies in presenting a versatile methodology for the systematic selection of favorable clones from extensive directed evolution libraries, which, in itself, constitutes a significant advancement.**

**The exhaustive characterization of Cas12f-ABE variants, as well as a comprehensive analysis of the recombinase clones as described, presents a challenge within the confines of a single manuscript due to inherent space limitations. While we recognize the value of such a detailed characterization, it's worth emphasizing that our primary intention was to introduce and demonstrate the efficacy of DEQSeq.**

**Nevertheless, to provide meaningful insights into the capabilities of DEQSeq, we undertook a targeted approach. We specifically benchmarked the TadA domains of three selected variants against the established ABE8e, and delved into a thorough comparison of the off-target activity exhibited by one variant, ABE3030, as compared to ABE8e. The results of these analyses serve as a robust endorsement of the effectiveness of DEQSeq, reinforcing its potential as a valuable tool to advance the field.**

**We also would like to point out, that we also demonstrate the effectiveness of DEQSeq with the recombinase variants that we characterize, including their off-target activity.**

**In conclusion, while we recognize the importance of a more extensive characterization, the current manuscript emphasizes the novel approach of DEQSeq and provides substantial evidence of its utility through focused assessments of specific variants. Further studies will have to explore the broader landscape of variant characterization, but our work contributes significantly to advancing the field by introducing and validating DEQSeq as a powerful methodology.**

4.      Could the authors consider inserting an enzyme cleavage site between the two loxF8 site and digesting with this enzyme prior to subcloning the 3 other target backbones (HG1, HG2, HG2L)? This could potentially help exclude variants that are not recombinantly active on the loxF8 site.
Minor points:

**Reply: It is indeed possible to perform this selection before cloning the subsampled library into the off-targets (see Suppl. Fig. 3). However, most of the recombinases in the pool already have good activity on loxF8 so that this enrichment would not substantially change the number of effective variants screened. In addition, the low number of inactive clones on loxF8 can also serve as an additional internal control, because none of these variants should recombine the off-target sites (which they don’t).**

Minor points:

1.      Please add statistical methods in Fig. 1e and Fig. 2e.

**Reply: As requested, we have added statistical analyses in Fig. 1e and Fig. 2e (now Fig. 2d and Fig. 4b, respectively).**

2.      In Fig.1 and 2, before transformed to E. coli, the plasmid library diagram missed the loxF8 site and promoter, target site of 3 sgRNA and sgRNA.

**A: We thank the reviewer for pointing this out. We have added these components to the plasmid diagrams in Fig. 1 and 2 (now Fig. 4).**

3.      The format of reference 15 should remain consistent with others.

**Reply: Formatting of reference 15 has been adjusted, thank you.**

Reviewer #2: This is a very interesting study that reports a widely applicable method to evolve and engineer DNA recombination and engineering enzymes. The authors smartly combine the enzyme-coding sequence variance and the target in sequence so that they can be sequenced together with long-read nanopore sequencing. Their platform was demonstrated with engineering designer recombinases and Un1Cas12f1-ABE systems.

**Reply: We thank reviewer#2 for the concise summary and positive overall evaluation.**

Major points:

1)      The manuscript is too succinct to follow. The rationale and design are hidden in the Figure legends and Methods. This is not the best way to write a paper. Based on the content, it would be nicer to write it as a full-length research article.

**Reply: We agree with the assessment of reviewer#2. Based on this recommendation, we have extended the manuscript to fulfill the criteria of a full-length research article.**

2)      In both cases (designer recombinase and Un1Cas12f1-ABE), what's the rationale for picking the three variants each? Was that truly random? It would be valuable to pick more clones for validation so that we can learn how reliable the system is and what kind of mutations are behind the improved activity.

**Reply: We appreciate this request and have expanded the analysis to six variants, each. Furthermore, the rationale for choosing these variants is now provided in the text. We also present the mutations for these additional variants in Suppl. Figs. 2 and 6, revealing what kind of mutations are behind the measured activities.**

3)      The authors listed protein alignments of the three highly efficient Un1Cas12f-ABE systems. Please provide full protein sequences and annotated vector sequences for readers to extract such information. Also, I suggest depositing these vectors to Addgene because many would be interested in testing and applying these improved ABEs in their organisms of interest.

**Reply: We thank reviewer#2 for this suggestion. We are now providing full protein sequences as Suppl. Table 3 and a detailed vector map of pCaSLIDE-ABE as Suppl. Fig. 8. As suggested, we will deposit the vectors to Addgene.**

4)      Will the system work well with larger enzymes and hence longer coding sequences? What will be the challenges in this case? Some interesting discussion on this point and others would make this paper more interesting.

**Reply: We appreciate this question from reviewer#2. We are very confident that the approach will also work with larger enzymes. In fact, we have already used the system for plasmids of sizes bigger then 10kb, where the evolved genome editing enzyme is encoded in more than 6kb. The method of nanopore sequencing provides almost unlimited sequence length. The only limitation is the size of the plasmid. We have extended our discussion to cover this point.**

5) Will the evolved work well in eukaryotic cells? Validation on this will add much to this story.

**Reply: Although the focus of the current manuscript is on the presentation of DEQSeq as a versatile methodology for the systematic selection of favorable clones from extensive directed evolution libraries, we acknowledge that a validation of variants in mammalian cells would add to the story. To address this point, we have tested three of the recombinase variants (now shown in Fig. 2e-g) and three of the TadA variants (now shown in Fig. 5 f,g and Suppl. Fig. 7c) in human tissue culture cells. The obtained results support the utility of DEQSeq to nominate favorable genome editing variants from large directed evolution libraries.**

Minor point:

1)      Page 5 line 64-69: Please mention the 1244 clone as this one showed reduced off-target effects without compromising the on-target recombination activity.

**Reply: We thank reviewer #2 for this suggestion. We now highlight variant #1244 in the text on page 5 as a promising candidate.**

**Second round of review**

**Reviewer 1**

The authors have addressed all of my previous concerns. Overall, the data is more detailed and portrays a very complete study now. I have one additional question more for the author, which perhaps could motivate the author to include an additional statement on the future potential of their method. Can DEQSeq only work on the basis of the SliDE plasmid? Along this line, could the authors propose major challenges that need to be addressed when using their approach with other selection methods.

**Reviewer 2**

The authors have diligently addressed the concerns raised. Their effort in enhancing the manuscript is evident through the inclusion of more background, expanded discussions, and more details for the experiment design section. These improvements elevate the manuscript, aligning it more closely with the standards of a research article and making it more accessible for readers. Furthermore, the restructured figures and the incorporation of additional data strengthen its suitability for publication in Genome Biology.

However, a few aspects still require attention:

1. There are some differences in the procedure of Nanopore sequencing and processing of loxF8 and Cas12f-ABE screen libraries. For instance, the use of the ligation sequencing kit and sequencing reads filter vary. Could this be attributed to the specific objectives of each library screen?

2. Line 405 and 406, please list all the specific reverse primers of UMI of the cluster of interest.

3. Line 410, “BsrgI” should be replaced by “BsrGI”.

4. In Supplementary Figure 7, the “Note the only synonymous bystander edit at position 12 “A” with sgRNA1 is indicated by a blue arrow” should be replaced by “Note the only synonymous bystander edit at position 12 “A” with sgRNA2 is indicated by a blue arrow”. Moreover, there's an inconsistency between the sgRNA name in this figure and what's presented in Supplementary Table 2.

5. The Methods section would benefit from the inclusion of vector cloning details for spABE8e and spABE3030.

**Authors Response**

**Point-by-point responses to the reviewers’ comments:**

Reviewer #1: The authors have addressed all of my previous concerns. Overall, the data is more detailed and portrays a very complete study now. I have one additional question more for the author, which perhaps could motivate the author to include an additional statement on the future potential of their method. Can DEQSeq only work on the basis of the SliDE plasmid? Along this line, could the authors propose major challenges that need to be addressed when using their approach with other selection methods.

Reply: We thank reviewer #1 for this positive feedback and the additional question. DEQSeq is not restricted to the SLiDE plasmid and can in principle be used with any plasmid containing the enzyme gene, UMI and target sequence. Other selection methods, such as PACE (PMID: 21478873) should also be suitable for use with DEQSeq. We have added the following sentence to the discussion to address this point: “Importantly, DEQSeq can be used with other plasmid designs and directed evolution strategies, making it very versatile.”

Reviewer #2: The authors have diligently addressed the concerns raised. Their effort in enhancing the manuscript is evident through the inclusion of more background, expanded discussions, and more details for the experiment design section. These improvements elevate the manuscript, aligning it more closely with the standards of a research article and making it more accessible for readers. Furthermore, the restructured figures and the incorporation of additional data strengthen its suitability for publication in Genome Biology.

Reply: We agree with the reviewer that the manuscript has been improved considerably and we thank the reviewer for the additional input, which we have addressed as outlined below.

However, a few aspects still require attention:

1. There are some differences in the procedure of Nanopore sequencing and processing of loxF8 and Cas12f-ABE screen libraries. For instance, the use of the ligation sequencing kit and sequencing reads filter vary. Could this be attributed to the specific objectives of each library screen?

Reply: The reason for using a different ligation sequencing kit was the Oxford Nanopore Technologies update during the course of the experiments. The older generation of the nanopore sequencing kit LSK110 delivers a raw read accuracy of ~92%, which was sufficient to obtain reliable results as presented. The accuracy of the newer kit LSK112 delivers a raw read accuracy of ~98-99%, which further improved the quality of the data. Because Oxford Nanopore Technologies keeps updating their sequencing technology, future users of DEQSeq will benefit from these improvements.

To adjust for the size of the sequenced fragments and the required quality, the filtering criteria were adjusted to deliver the best output for each screen.

2. Line 405 and 406, please list all the specific reverse primers of UMI of the cluster of interest.

Reply: We thank the reviewer for mentioning the missing primers, we added the names of the primers to the text.

3. Line 410, “BsrgI” should be replaced by “BsrGI”.

Reply: We corrected the restriction site name to BsrGI in all occurrences in the text – thank you.

4. In Supplementary Figure 7, the “Note the only synonymous bystander edit at position 12 “A” with sgRNA1 is indicated by a blue arrow” should be replaced by “Note the only synonymous bystander edit at position 12 “A” with sgRNA2 is indicated by a blue arrow”. Moreover, there's an inconsistency between the sgRNA name in this figure and what's presented in Supplementary Table 2.

Reply: We apologize for the inconsistency; we corrected all references of sgRNA1 and sgRNA2 to be consistent.

5. The Methods section would benefit from the inclusion of vector cloning details for spABE8e and spABE3030.

Reply: We agree with this assessment and the vector cloning details have now been added.
